# Determinants of Serum Biotin (Vitamin B7) Concentrations: Influence of Age, Sex, and Season in a Large Population Cohort: A Retrospective Cross-Sectional Study

**DOI:** 10.3390/biomedicines14102320

**Published:** 2026-10-13

**Authors:** Esraa Almalki, Muhannad Alharthi, Mashael J. Abu Alola, Albaraa Alharbi, Osamah T. Khojah, Saltana A. Alhowaiti, Abdullah Al Marzan, Khalid M. Sumaily, Ahmed A. Al-Qahtani, Khalid M. Al-Qahtani

**Affiliations:** 1 Advanced Biochemistry Center, Medical Diagnostic Laboratories (MDLAB), Dr. Sulaiman Al-Habib Medical Group, Riyadh 13321, Saudi Arabia; almalkiesra@gmail.com (E.A.); imuhannadx@gmail.com (M.A.); albaraa04@gmail.com (A.A.); 2 Department of Infection and Immunity, Research Centre, King Faisal Specialist Hospital and Research Centre, Riyadh 11211, Saudi Arabia; malsaeedi@kfshrc.edu.sa (M.J.A.A.); salhuwaiti@kfshrc.edu.sa (S.A.A.); 3 Department of Pathology, College of Medicine, King Saud University, Riyadh 11472, Saudi Arabia; asd@ksu.edu.sa (O.T.K.); khaled.asumaily@drsulaimanalhabib.com (K.M.S.); 4 CMO Office, Medical Diagnostic Laboratories (MDLAB), Dr. Suliman Al-Habib Medical Group, Riaydh 13321, Saudi Arabia; 5 Department of Biochemistry and Molecular Biology, Shahjalal University of Science and Technology, Sylhet 3114, Bangladesh; marzansust16@gmail.com; 6 Department of Data Science, Toxicology Society of Bangladesh, Dhaka 1000, Bangladesh; 7 Department of Microbiology, College of Medicine, Alfaisal University, Riyadh 11533, Saudi Arabia

**Keywords:** biotin, vitamin B7, micronutrient status, seasonal variation, Saudi Arabia, cross-sectional study, nutritional epidemiology

## Abstract

**Background/Objectives**: Population-level data on serum biotin (vitamin B7), an essential carboxylase cofactor, remain limited, particularly in Middle Eastern populations. We described serum biotin distribution and examined demographic and seasonal correlates in a large Saudi cohort. **Methods**: This retrospective cross-sectional study included 11,735 adults (79.0% female; mean age 36.5 ± 11.8 years) with serum biotin measurements from an institutionally supported outpatient clinical/laboratory testing program in Riyadh. Serum biotin was measured by competitive enzyme-linked immunosorbent assay (ELISA); results above the measuring range were recorded at its upper limit (1100 ng/L). Associations were evaluated using non-parametric tests and multivariable regression, including regression for censored data. **Results**: Mean serum biotin was 351.9 ± 251.8 ng/L (median: 270.9 ng/L). By concentration band, 0.5% of the results were <100 ng/L, 42.4% were 100–250 ng/L, 50.5% were >250 to <1100 ng/L, and 6.6% were at the upper limit (≥1100 ng/L); excluding the latter, the mean was 299.2 ± 160.2 ng/L. Age showed a weak positive correlation with biotin (ρ = 0.18, *p* < 0.001). Female sex was associated with higher odds of a result at the upper limit (odds ratio [OR] = 1.93, 95% confidence interval [CI]: 1.55–2.40, *p* < 0.001), whereas the median concentration was slightly higher in males (287.9 vs. 266.8 ng/L). In the complete calendar year 2024, the seasonal medians were 243.2, 290.7, 308.5, and 262.9 ng/L in winter, spring, summer, and autumn, respectively; this pattern was not reproduced in the first half of 2025. **Conclusions**: In this large Saudi outpatient cohort, concentrations below 100 ng/L were uncommon, whereas concentrations of 100–250 ng/L were frequent and 6.6% of results reached the upper limit of the assay. Age, sex, nationality, and season were associated with serum biotin, but findings require cautious interpretation because of the retrospective design, clinically requested testing, and absence of key clinical and behavioral covariates. These data provide descriptive information and support future studies incorporating supplementation history, renal function, clinical indication for testing, and functional biomarkers of biotin status.

## 1. Introduction

Biotin (vitamin B7), a water-soluble B-complex vitamin, serves as an essential cofactor for five carboxylase enzymes involved in critical metabolic pathways, including gluconeogenesis, fatty acid synthesis, and amino acid catabolism [1,2]. Beyond its well-established role in cellular metabolism, biotin has gained increasing attention for its involvement in gene regulation, cell signaling, and the maintenance of chromatin structure through covalent modification of histone proteins [3,4]. Adequate biotin status is essential for maintaining healthy skin, hair, and nails, and deficiency states have been associated with dermatological manifestations, including alopecia and characteristic skin rashes, as well as neurological symptoms such as depression, lethargy, paresthesia, and in severe cases, seizures and cognitive impairment [5,6,7].

Despite biotin’s fundamental importance, population-level data characterizing normal serum biotin concentrations and identifying factors that influence biotin status remain limited, particularly from Middle Eastern populations. The majority of existing biotin studies have focused on clinical deficiency states, such as biotinidase deficiency, or on supplementation trials in Western populations, with few large-scale observational studies examining the distribution and determinants of serum biotin in diverse community settings [8,9]. Understanding the factors that influence biotin concentrations is clinically relevant, as biotin deficiency may be underrecognized in adults, and several conditions, including pregnancy, prolonged antibiotic therapy, inflammatory bowel disease, and chronic alcohol consumption, can impair biotin status [10,11].

Several factors have been proposed to influence serum biotin concentrations, including demographic characteristics such as age and sex, genetic variations affecting biotin metabolism, dietary intake patterns, seasonal variations in dietary habits, and the presence of underlying health conditions [5]. However, the relative contribution of these factors and their interactions remain poorly characterized in large population studies. Age-related changes in nutrient absorption, metabolic rate, and dietary patterns may affect biotin status throughout the lifespan, while sex differences in body composition, hormonal status, and nutritional requirements may influence circulating biotin levels [12]. Additionally, seasonal variations in food availability, dietary diversity, and vitamin intake patterns, particularly relevant in regions with distinct climatic seasons, may contribute to temporal fluctuations in biotin status [13,14].

The Kingdom of Saudi Arabia, with its unique demographic composition, dietary practices influenced by both traditional and Westernized patterns, and distinct seasonal climate variations, provides an important context for examining biotin status [15]. The region’s population includes predominantly Saudi nationals alongside diverse expatriate communities from various countries, potentially introducing heterogeneity in genetic backgrounds, dietary habits, and nutritional status. Previous nutritional studies in the Gulf region have identified specific patterns of micronutrient deficiencies and unique nutritional challenges related to rapid dietary transitions and lifestyle changes, though comprehensive data on biotin status remains scarce [16]. From an epidemiological perspective, characterizing this poorly described micronutrient biomarker in a Middle Eastern clinical setting may help define regional evidence gaps and priorities for prospective nutritional surveillance.

Serum biotin concentrations can be summarized using predefined concentration bands to describe population-level distribution patterns. However, serum biotin cutoffs are not universally standardized, and serum concentration alone does not establish clinical nutritional status [3]. Therefore, category-based findings in this study were interpreted descriptively, particularly in the context of increasing biotin supplementation use [17].

The present study aimed to describe serum biotin (vitamin B7) concentrations in a large Saudi adult cohort and to examine their associations with age, sex, nationality, and season. Specifically, we characterized the population distribution of serum biotin, quantified differences across demographic strata, and evaluated selected demographic correlates using multivariable regression. Given the retrospective cross-sectional design and limited covariate availability, these analyses were intended to characterize descriptive epidemiological patterns and associations rather than causal effects.

## 2. Materials and Methods

### 2.1. Study Design and Population

This retrospective cross-sectional study analyzed anonymized demographic and laboratory data generated within an institutionally supported outpatient clinical/laboratory testing program at Dr. Sulaiman Al Habib Medical Group, Riyadh, Saudi Arabia, between January 2024 and June 2025. These dates refer to the timing of clinical care and laboratory testing, not prospective research recruitment. A total of 11,735 participants were included in the analysis, based on anonymized data obtained from adults attending outpatient clinical/laboratory assessment services within Dr. Sulaiman Al Habib Medical Group. Routine health assessments comprised general outpatient evaluations, preventive health screenings, and follow-up visits for stable chronic conditions such as diabetes, hypertension, and hyperlipidemia, and did not include specialized pregnancy-related clinics, rehabilitation settings, or acute emergency care. No participants were recruited, contacted, or tested for research purposes. All clinical records, biospecimen collection, and laboratory data analyzed in this study were generated within Dr. Sulaiman Al Habib Medical Group. Post-data-generation analysis of the anonymized dataset, scientific interpretation, and manuscript development were conducted in collaboration with King Faisal Specialist Hospital and Research Centre. The reason each serum biotin test was requested was not captured in the research dataset; the cohort therefore represents adults who underwent serum biotin testing in routine care and is not a probability sample of the population served by the institution.

Inclusion criteria included adults aged ≥ 18 years with available serum samples for biotin analysis. Exclusion criteria included records lacking the variables required for the planned analyses, insufficient sample volume for analysis, or known conditions requiring high-dose biotin supplementation (e.g., biotinidase deficiency). Individuals with known conditions affecting nutrient absorption (e.g., inflammatory bowel disease, celiac disease, or other malabsorption syndromes) were excluded where documented in clinical records. Use of over-the-counter biotin-containing supplements was not recorded and could not be applied as an exclusion criterion.

The retrospective research use of the anonymized clinical and laboratory data analyzed in this study was approved by the Institutional Review Board (IRB) of Dr. Sulaiman Al Habib Medical Group (RC25.12.119/2025). The requirement for informed consent was waived by the IRB because the study involved secondary analysis of fully anonymized retrospective data, with no direct patient contact, no prospective research recruitment, and no access to identifiable patient information by the research team. All data were anonymized before analysis in accordance with institutional ethical requirements. Laboratory measurements, including serum biotin, had already been generated within the clinical/laboratory care setting and were not performed prospectively for research purposes. No additional laboratory testing was performed solely for this retrospective analysis. The serum biotin measurements and related laboratory processes included in this retrospective dataset were institutionally supported and no research-specific testing costs were charged to participants. The anonymized research dataset available for this analysis was limited to age, sex, nationality, serum biotin concentration, and date of sample collection. Detailed clinical indication for testing, dietary habits, lifestyle factors, smoking, alcohol use, supplementation, and broader clinical diagnoses were not available for this cohort and therefore could not be included in the adjusted analyses.

### 2.2. Data Collection

Demographic information, including age, sex, and nationality, was obtained from institutional clinical records maintained by Dr. Sulaiman Al Habib Medical Group. For this retrospective analysis, sex refers to the binary male/female category documented in the institutional clinical record system; information on gender identity was not available. Sex was not recorded for one participant, who was included in the overall analyses and excluded from analyses by sex. Blood samples were collected following standard phlebotomy procedures after an overnight fast (minimum 8 h). Samples were allowed to clot at room temperature for 30 min, then centrifuged at 3000× *g* for 10 min. Serum was separated, aliquoted into cryovials, and stored at −20 °C until analysis. All samples were analyzed within 6 months of collection. The month and season of sample collection were recorded for each participant to enable analysis of seasonal variation. No structured research questionnaires were used, and no additional patient-reported data were collected for the purpose of this study.

### 2.3. Serum Biotin Measurement

Serum biotin (vitamin B7) concentrations were determined by enzyme-linked immunosorbent assay (ELISA) using the IDK^®^ Biotin ELISA kit (Immundiagnostik AG, Bensheim, Germany; catalog number K 8141) according to the manufacturer’s instructions. Briefly, 50 μL of standards, controls, or samples plus 50 μL sample dilution buffer was added to streptavidin-coated wells and incubated for 30 min at room temperature (20–30 °C). After washing 5 times with diluted wash buffer (250 μL/well), 100 μL enzyme-labeled biotin conjugate was added and incubated for 30 min. Following another wash step, 100 μL tetramethylbenzidine substrate was added and incubated for 8–12 min in the dark. The reaction was stopped with 100 μL diluted sulfuric acid, causing a color change from blue to yellow. Absorbance was measured immediately at 450 nm (reference: 620 nm) using a microtiter plate reader BioTek Synergy HTX (Agilent, Santa Clara, CA, USA).

### 2.4. Quality Control and Calibration

Each assay plate included a six-point calibration curve using the provided ready-to-use standards (STDs) with concentrations ranging from 0 to 1100 ng/L as specified in the kit specification sheet. Two quality control samples provided by the manufacturer (CTRL A and CTRL B) with known biotin concentration ranges were analyzed in duplicate on each plate to verify assay performance. Additionally, an internal pooled serum control prepared from leftover patient samples was included on each plate to monitor plate-to-plate variability and assess inter-assay precision throughout the study period. Control values were required to fall within the manufacturer’s specified ranges (±2 standard deviations [SD] from target values) for the assay run to be accepted. Any plate failing quality control criteria was repeated. Plate, run, and kit-lot identifiers were not available in the anonymized research dataset.

### 2.5. Data Analysis and Calculation

Standard curves were generated using four-parameter logistic (4-PL) regression, plotting optical density (absorbance at 450 nm minus reference at 620 nm) against biotin concentration. Sample concentrations were interpolated from the standard curve using Python (version 3.11.5; SciPy v1.11, NumPy v1.25). For duplicate measurements, the mean value was used if the coefficient of variation (CV) was ≤15%; samples with CV > 15% were re-analyzed. Results above the upper limit of quantification were recorded at that limit (1100 ng/L); post-dilution concentrations were not available for analysis, and these results were therefore treated as right-censored, that is, the true concentration is known only to be at or above 1100 ng/L. Two results slightly above the highest calibrator were retained as reported (1160.0 and 1176.3 ng/L). Values below the limit of quantitation (LoQ) were included for descriptive purposes but interpreted with caution.

The assay performance characteristics reported by the manufacturer were: limit of blank (LoB) of 25 ng/L, limit of detection (LoD) of 32.4 ng/L, limit of quantitation (LoQ) of 48.1 ng/L, measuring range of 48.1–1100 ng/L, intra-assay precision (CV) of 6.0–6.7% (*n* = 40), inter-assay precision (CV) of 4.8–10.9% (*n* = 15), recovery of 89.6–115.2%, and linearity of 63.3–1134.8 ng/L (non-linearity < ±20%). Values below the LoQ were retained in the dataset for descriptive reporting and classification but were interpreted cautiously because they fall below the validated quantitation threshold.

### 2.6. Definition of Serum Biotin Concentration Bands

Participants were categorized into four predefined serum biotin concentration bands adapted from the prior literature and laboratory guidance: <100 ng/L, 100–250 ng/L, >250 to <1100 ng/L, and ≥1100 ng/L [1,8]. The two lower thresholds are the interpretive ranges given in the manufacturer’s instructions for use (<100 ng/L, vitamin deficiency; 100–250 ng/L, suboptimal status; >250 ng/L, healthy); the upper band corresponds to the upper limit of the measuring range, contains the right-censored results, and is not a biological threshold. In the tables and figures, the bands are referred to by their concentration ranges and not by clinical labels. These bands were used to summarize the distribution of serum biotin concentrations in the cohort. Because the bands were derived directly from the continuous serum biotin measurement, they were interpreted descriptively and were not considered independent clinical diagnoses of nutritional status. Clinical interpretation of these bands would require additional information, including functional biomarkers such as urinary 3-hydroxyisovaleric acid excretion and carboxylase activity [1,8,18]. The thresholds are not internationally standardized, and other sources use different values, for example, 400 ng/L as the lower limit of an optimal concentration [19]; band frequencies therefore depend on the thresholds chosen.

### 2.7. Seasonal Classification

Sample collection dates were categorized into four meteorological seasons: winter (December–February), spring (March–May), summer (June–August), and autumn (September–November). Monthly analyses were also performed to capture granular temporal patterns. Because the study window covered 18 months (January 2024 to June 2025), the calendar months January to June were sampled in two years and July to December in one. Seasonal analyses were therefore based primarily on the complete calendar year 2024.

### 2.8. Statistical Analysis

All statistical analyses were performed using Python (version 3.11.5) with relevant packages for statistical computing and visualization. Analysis scripts are available upon request.

Descriptive statistics are presented as mean ± SD and median (interquartile range [IQR]) for continuous variables, and frequencies (percentages) for categorical variables. Because results above the measuring range were recorded at its upper limit (right-censored), medians and rank-based tests were used for the primary comparisons, means include censored results at the recorded value, and a sensitivity analysis excluded results at the upper limit. Between-group comparisons used Mann–Whitney U tests for two-group comparisons and Kruskal–Wallis H tests for multiple-group comparisons, with Bonferroni-corrected post-hoc pairwise comparisons where applicable. Associations between biotin and age were assessed primarily using Spearman’s correlation (ρ), which makes no distributional assumption and is robust to the ceiling at the upper limit; Pearson correlation (r) on log-transformed data was computed as a secondary sensitivity analysis, with locally weighted scatterplot smoothing (LOWESS) for trend visualization. Two-way analysis of variance (ANOVA) examined the effects of age group and sex on log-transformed biotin concentrations. Comparisons between Saudi participants and each non-Saudi nationality with at least 25 participants were adjusted for multiplicity using the Holm method.

As secondary analyses, serum biotin concentration bands were also examined to summarize the distributional patterns across the cohort. The distribution of concentration bands across age groups was evaluated using a chi-square test of independence and visualized using standardized residual heatmaps. Because the bands are derived from the same underlying continuous serum biotin measure, concentrations were not compared statistically across bands; instead, the dependence of band frequencies on the choice of threshold was examined using the empirical cumulative distribution.

Binary logistic regression identified factors associated with a serum biotin result at the upper limit of the measuring range (≥1100 ng/L), with results reported as odds ratios (ORs) and 95% confidence intervals (CIs). Model covariate selection was restricted to variables available in the anonymized dataset. Multivariable linear regression assessed the associations of age, sex, and nationality (non-Saudi vs. Saudi) with serum biotin concentration as a continuous outcome. Because results at the upper limit are right-censored, the primary linear model was a censored (Tobit) regression of log-transformed concentration, with coefficients expressed as percentage differences; ordinary least-squares models of recorded values, with and without results at the upper limit, and median regression of log-transformed concentration, which is unaffected by censoring, were fitted as sensitivity analyses. Sex-specific models, an age-by-sex interaction term, and restricted cubic splines for age were used to examine effect modification and nonlinearity. In the 2024 data, seasonal differences were examined in censored regression models adjusted for age, sex, and nationality, from which covariate-adjusted geometric means with 95% CIs were derived, and within subgroups defined by sex, age, and nationality. In the pooled 18-month data, models were additionally adjusted for calendar year, and temporal stability was examined by identifying change points in the weekly median concentration and entering the resulting periods as a covariate in place of calendar year (Supplementary Figure S1). The possible magnitude of the right-censored results was estimated under two models: (A) the upper tail of the censored log-normal regression, and (B) continuation of the density observed between 600 and 1100 ng/L above the limit as an exponential tail, fitted separately by sex. These model-based estimates are not measured values. Model assumptions were evaluated using standard diagnostic procedures, including residual plots and variance inflation factors. Statistical significance was set at α = 0.05 (two-tailed).

## 3. Results

### 3.1. Study Population Characteristics

A total of 11,735 participants were included in this cross-sectional study. The cohort comprised 9270 females (79.0%) and 2464 males (21.0%); sex was not recorded for one participant (<0.1%), which accounts for the total of 11,735. A numeric serum biotin result was available for 11,734 participants. The mean age was 36.5 ± 11.8 years (median: 34 years; range: 18–100 years) (Table 1). The majority of participants were Saudi nationals (*n* = 10,778, 91.8%), while non-Saudi participants accounted for 8.2% (*n* = 957) of the cohort. Non-Saudi participants were recorded under 89 nationality labels, most frequently Jordanian (*n* = 149), Egyptian (*n* = 115), Indian (*n* = 75), Yemeni (*n* = 69), and Syrian (*n* = 64); the number of participants in every category is given in Supplementary Table S1.

The mean serum biotin (vitamin B7) concentration was 351.9 ± 251.8 ng/L (median = 270.9 ng/L; IQR: 200.8–388.3; range: 38.3–1176.3 ng/L). Results were grouped into four predefined serum biotin concentration bands: <100 ng/L (*n* = 62; 0.5%), 100–250 ng/L (*n* = 4977; 42.4%), >250 to <1100 ng/L (*n* = 5923; 50.5%), and ≥1100 ng/L (*n* = 772; 6.6%). The mean (±SD) serum concentrations in these bands were 80.73 ± 14.53 ng/L, 187.43 ± 40.32 ng/L, 395.48 ± 160.91 ng/L, and 1100.18 ± 3.49 ng/L, respectively. Of the 772 results in the highest band, 770 were recorded as exactly 1100 ng/L, the upper limit of the measuring range, and two were slightly higher (1160.0 and 1176.3 ng/L); these results are right-censored, and their true concentrations are unknown. Excluding them, the mean was 299.2 ± 160.2 ng/L and the median was 261.0 ng/L (IQR: 196.1–355.3; *n* = 10,962). The median and quartiles of the whole cohort are not affected by censoring, because 93.4% of results lie below the upper limit.

Table 1 shows the demographic and biochemical characteristics of the study cohort (*n* = 11,735). Section A summarizes the overall cohort age and serum biotin concentrations. Section B provides detailed age percentiles. Section C presents sex and nationality distributions. Section D displays serum biotin concentration bands with counts, percentages, and descriptive statistics. Values are presented as mean ± SD or median (IQR) where appropriate.

### 3.2. Distribution of Serum Biotin Concentrations

**Figure 1:**
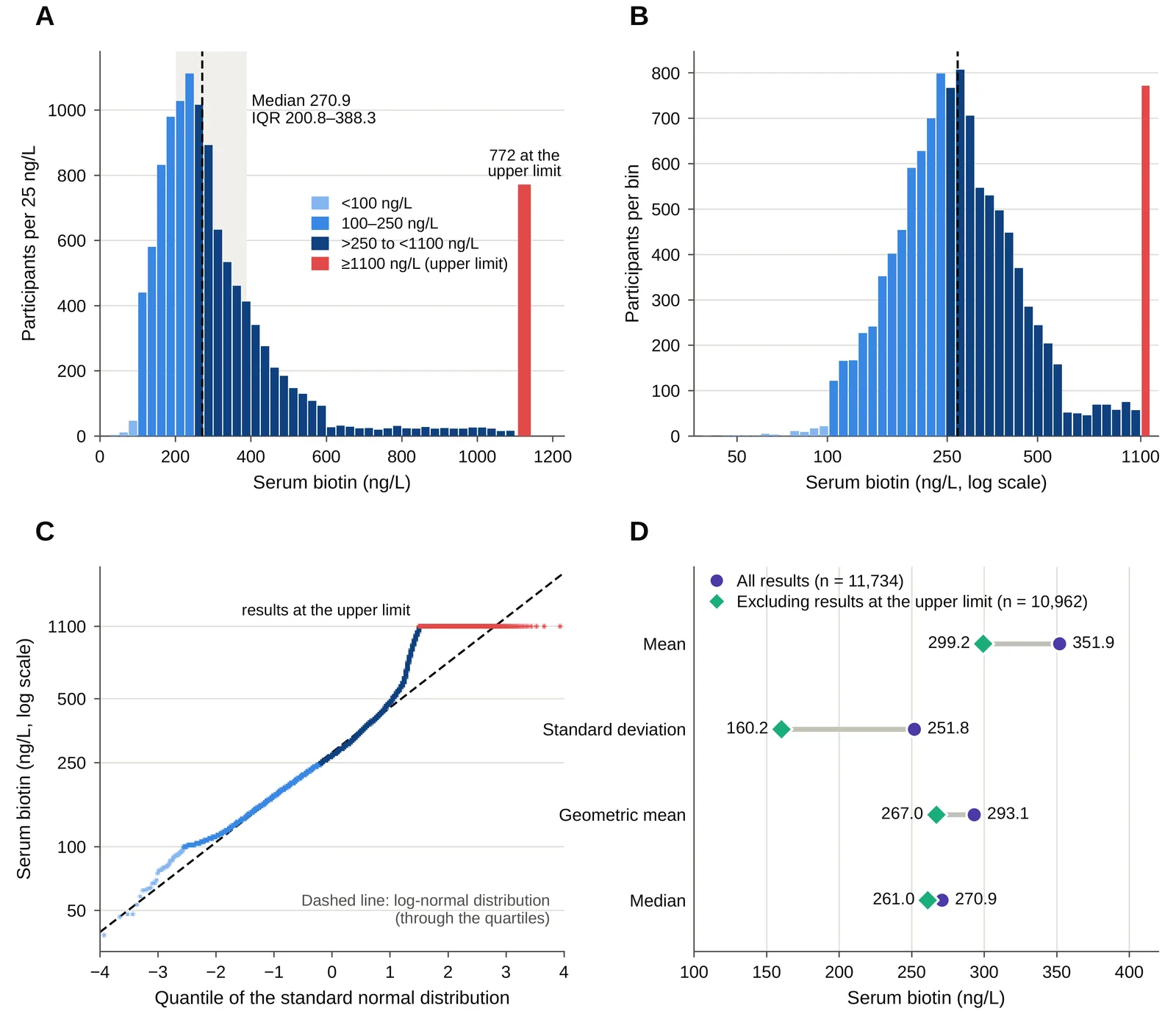
Distributionof serum biotin concentrations in the study cohort (*n* = 11,734). (**A**) Histogram of serum biotin concentrations (ng/L), showing a right-skewed distribution; bars are colored by concentration band, the dashed line marks the median, and the shaded area marks the interquartile range. The red bar contains the 772 results recorded at the upper limit of the measuring range (≥1100 ng/L; right-censored). (**B**) Histogram of the same concentrations on a log_10_ scale. (**C**) Normal quantile–quantile plot of log-transformed concentrations; the dashed line shows a log-normal distribution fitted through the quartiles, and the horizontal segment is formed by the results at the upper limit. (**D**) Mean, standard deviation, geometric mean, and median for all results and after exclusion of the results at the upper limit. These findings supported the use of non-parametric methods and of regression for censored data in subsequent analyses.

The overall mean serum biotin concentration was 351.9 ± 251.8 ng/L, with a median of 270.9 ng/L (IQR: 200.8–388.3 ng/L; range: 38.3–1176.3 ng/L) (Figure 1). The distribution of serum biotin levels was right-skewed (Shapiro–Wilk W = 0.729, *p* < 0.001), with a discrete spike at 1100 ng/L formed by the results recorded at the upper limit of the measuring range (Figure 1A). Log_10_ transformation improved symmetry (W = 0.949, *p* < 0.001), although the spike remained (Figure 1B) and the upper part of the distribution departed from a log-normal distribution (Figure 1C). The mean and SD, but not the median, were sensitive to the results at the upper limit (Figure 1D). These features supported the use of non-parametric methods and of regression for censored data in subsequent analyses.

### 3.3. Serum Biotin Concentration Bands

**Figure 2:**
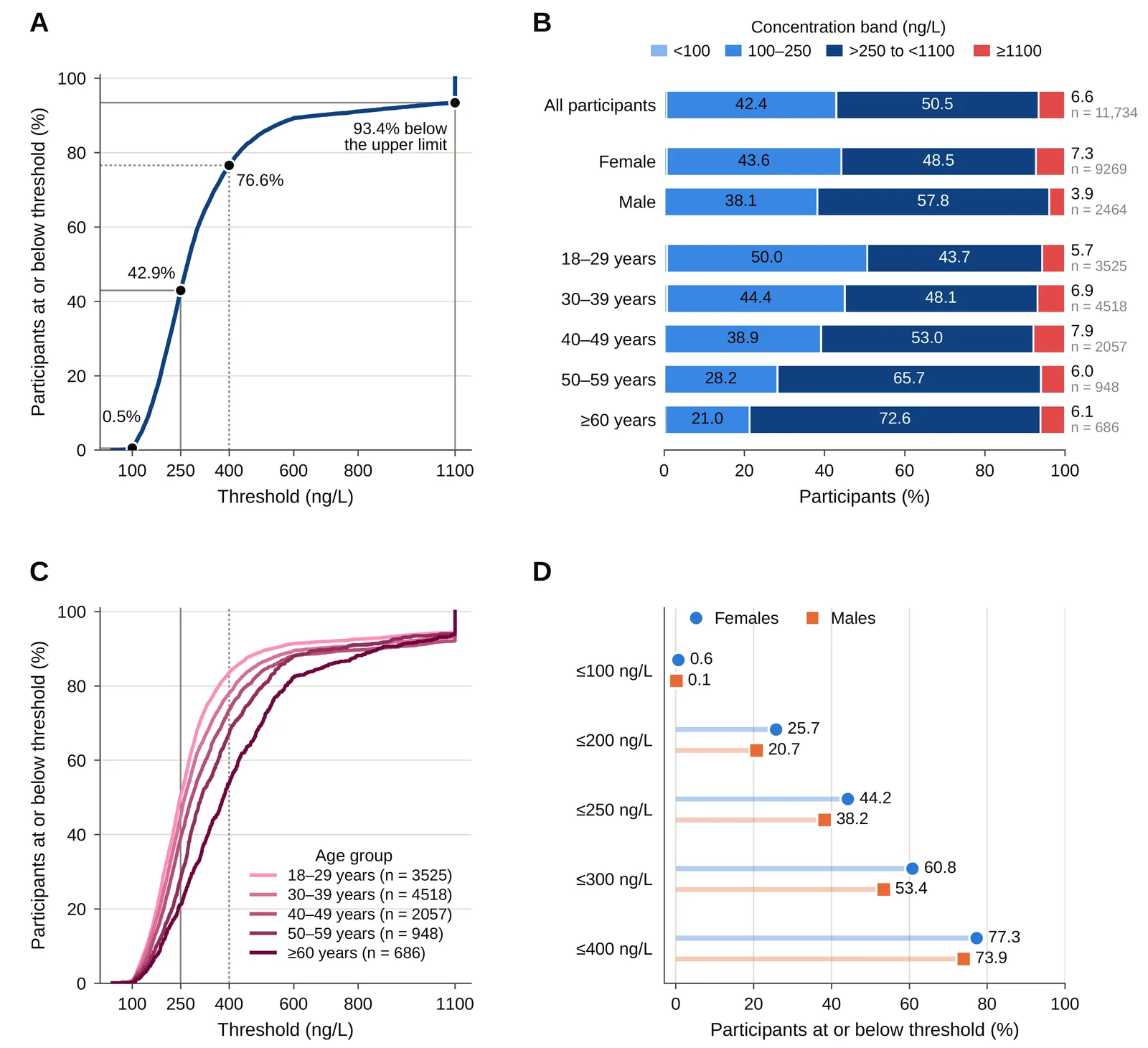
Serum biotin concentration bands and their dependence on the choice of threshold. (**A**) Cumulative distribution of serum biotin: the percentage of participants at or below each threshold is shown for the thresholds used in this study (100 and 250 ng/L; solid lines) and for an alternative threshold of 400 ng/L used elsewhere [19] (dotted line); 93.4% of results were below the upper limit of the measuring range (1100 ng/L). (**B**) Percentage of participants in each concentration band, overall and by sex and age group; the red band contains the results recorded at the upper limit of the measuring range (≥1100 ng/L), and its percentage is given to the right of each bar. (**C**) Cumulative distributions by age group; vertical lines mark 250 ng/L (solid) and 400 ng/L (dotted). (**D**) Percentage of females and males with a concentration at or below each of five thresholds. The bands are derived from the underlying continuous serum biotin measure and should be interpreted descriptively and not as diagnostic categories.

Results were grouped descriptively as <100 ng/L (*n* = 62, 0.5%), 100–250 ng/L (*n* = 4977, 42.4%), >250 to <1100 ng/L (*n* = 5923, 50.5%), and ≥1100 ng/L (*n* = 772, 6.6%). The median serum biotin concentrations in these bands were approximately 85.6, 191.7, 345.1, and 1100.0 ng/L, respectively (Table 1). Band frequencies depend directly on where the thresholds are placed (Figure 2A): 42.9% of results were at or below 250 ng/L, whereas 76.6% were below 400 ng/L, a threshold used elsewhere [19]. The proportion below a given threshold should therefore not be interpreted as the prevalence of inadequate biotin status. The distribution of bands by sex and age group is shown in Figure 2B and Table 2, cumulative distributions by age group in Figure 2C, and the percentages of females and males below alternative thresholds in Figure 2D. Because the bands are directly derived from serum biotin concentration, concentrations were not compared statistically across bands.

### 3.4. Sex Differences in Serum Biotin Concentrations

**Figure 3:**
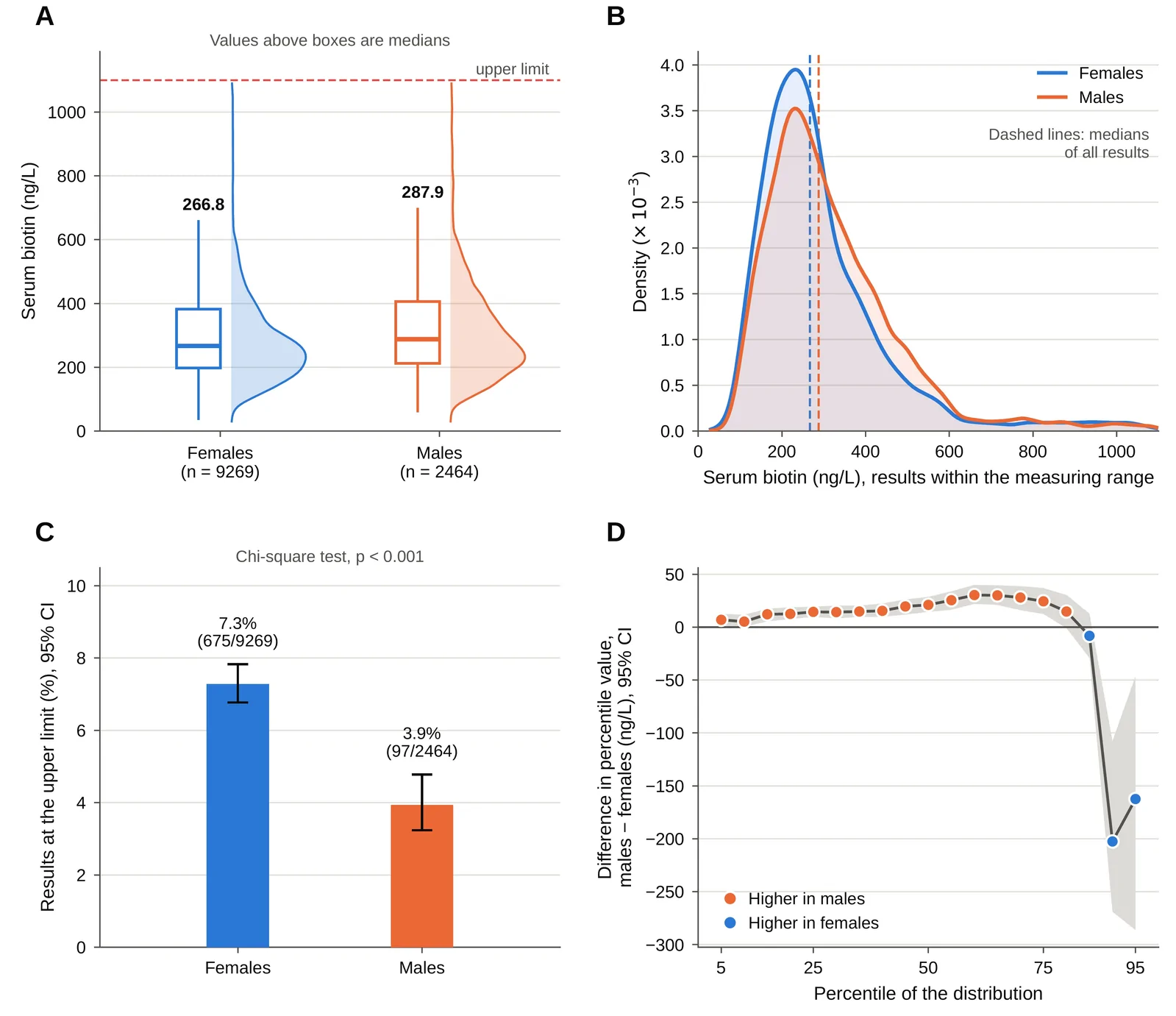
Sex differences in serum biotin concentrations. (**A**) Box plots with density curves for females and males; boxes show the interquartile range, the horizontal line the median (value shown), and whiskers the most extreme values within 1.5 × IQR; density curves are drawn for results within the measuring range, and the dashed line marks the upper limit of the measuring range. (**B**) Kernel density estimates of results within the measuring range by sex; dashed lines mark the medians of all results. (**C**) Percentage of results at the upper limit of the measuring range (≥1100 ng/L) by sex, with 95% confidence intervals (chi-square test, *p* < 0.001). (**D**) Difference between males and females in the value of each percentile of the distribution (5th to 95th), with bootstrap 95% confidence intervals; values above zero indicate higher concentrations in males. Differences between groups were statistically significant by Mann–Whitney U test (*p* < 0.001).

Serum biotin concentrations differed significantly between males and females (Mann–Whitney U, *p* < 0.001). Median B7 levels were slightly higher in males (287.9 ng/L; IQR: 212.4–406.3) than in females (266.8 ng/L; IQR: 198.0–381.8), although the interquartile ranges overlapped substantially (Figure 3A).

Kernel density estimation (KDE) analysis (Figure 3B) revealed similar primary peaks for both sexes at approximately 200–300 ng/L, consistent with the population’s central tendency. The male distribution was shifted slightly toward higher concentrations within the measuring range (Figure 3D). In contrast, results at the upper limit of the measuring range were more frequent in females (675/9269; 7.3%) than in males (97/2464; 3.9%; chi-square test, *p* < 0.001) (Figure 3C). Consequently, the mean recorded concentrations were similar in females and males (352.8 vs. 348.6 ng/L), whereas after exclusion of results at the upper limit the median was lower in females (256.3 ng/L) than in males (281.9 ng/L). These findings indicate modest sex-related differences in circulating biotin concentrations.

### 3.5. Association Between Age and Serum Biotin Levels

**Figure 4:**
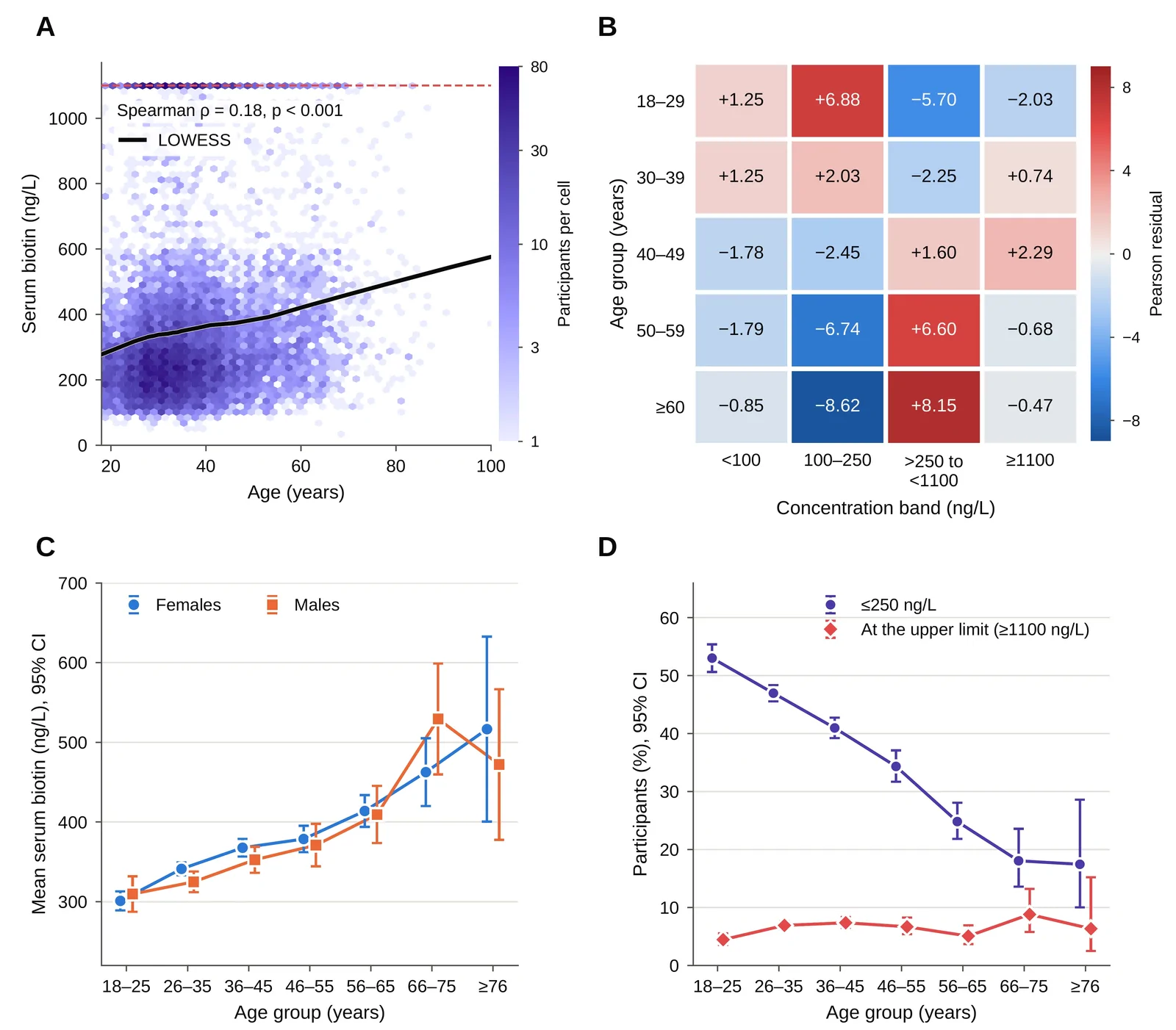
Age-related patterns in serum biotin concentrations. (**A**) Density plot (hexagonal bins) of serum biotin concentration against age, with LOWESS smoothing; the dashed line marks the upper limit of the measuring range. Spearman correlation (primary analysis; ρ = 0.18, *p* < 0.001) and Pearson correlation of log-transformed data (sensitivity analysis; r = 0.18, *p* < 0.001) showed weak positive associations. (**B**) Heatmap of standardized (Pearson) residuals from the chi-square analysis of age group by concentration band (*p* < 0.001); because the bands are derived from serum biotin concentration, this panel visualizes distributional patterns only. (**C**) Mean serum biotin concentration with 95% confidence interval by age group and sex; two-way ANOVA showed a significant main effect of age (*p* < 0.001), with no significant sex effect or age × sex interaction. (**D**) Percentage of participants with a concentration at or below 250 ng/L and with a result at the upper limit of the measuring range (≥1100 ng/L) by age group, with 95% confidence intervals. In panels C and D, participants aged 76 years or older are pooled. Means, SDs, and sample sizes by age group and sex are given in Supplementary Table S2.

As shown in Figure 4A, Spearman correlation analysis, the primary analysis, revealed a weak but statistically significant positive association between age and serum biotin (B7) concentration (ρ = 0.18, *p* < 0.001). Pearson correlation of log-transformed data, performed as a secondary sensitivity analysis, yielded similar results (r = 0.18, *p* < 0.001), indicating that serum biotin levels tend to increase modestly with advancing age. Consistent with this, the scatter plot with LOWESS smoothing demonstrated a slight upward trend across the age range, though substantial interindividual variability was evident.

Age-stratified analysis revealed progressively higher mean serum biotin concentrations from early adulthood through the 66–75-year age group. Mean serum biotin levels rose from 18–25 years (females: 301 ± 222 ng/L; males: 310 ± 208 ng/L) through 56–65 years (414 ± 241 ng/L; 409 ± 231 ng/L), and were highest in the oldest groups (482 ng/L at 66–75 years and 493 ng/L at 76–85 years); however, estimates in the oldest strata should be interpreted cautiously because of the limited sample size (*n* = 56 at 76–85 years). Two-way ANOVA confirmed a significant main effect of age (*p* < 0.001), whereas sex (*p* = 0.12) and the age × sex interaction (*p* = 0.65) were not significant, indicating similar age-related trends in females and males (Figure 4C; Supplementary Figure S2A; Supplementary Table S2). Figure 4B presents a descriptive heatmap of the distribution of serum biotin concentration bands across age groups. Younger adults were overrepresented in the 100–250 ng/L band, whereas older adults were overrepresented in the >250 to <1100 ng/L band; because these bands are derived from serum biotin itself, this panel is intended only to visualize age-related distribution patterns and not to imply independent predictive effects. The proportion of results at or below 250 ng/L fell from 53.0% at 18–25 years to 18.1% at 66–75 years, whereas the proportion of results at the upper limit of the measuring range showed no consistent trend with age (4.5–8.8% across age groups, with participants aged 76 years or older pooled; Figure 4D). The observed age association should be interpreted cautiously in light of unmeasured factors such as supplement exposure, renal function, and clinical indication for testing.

### 3.6. Nationality Differences in Serum Biotin Concentrations

**Figure 5:**
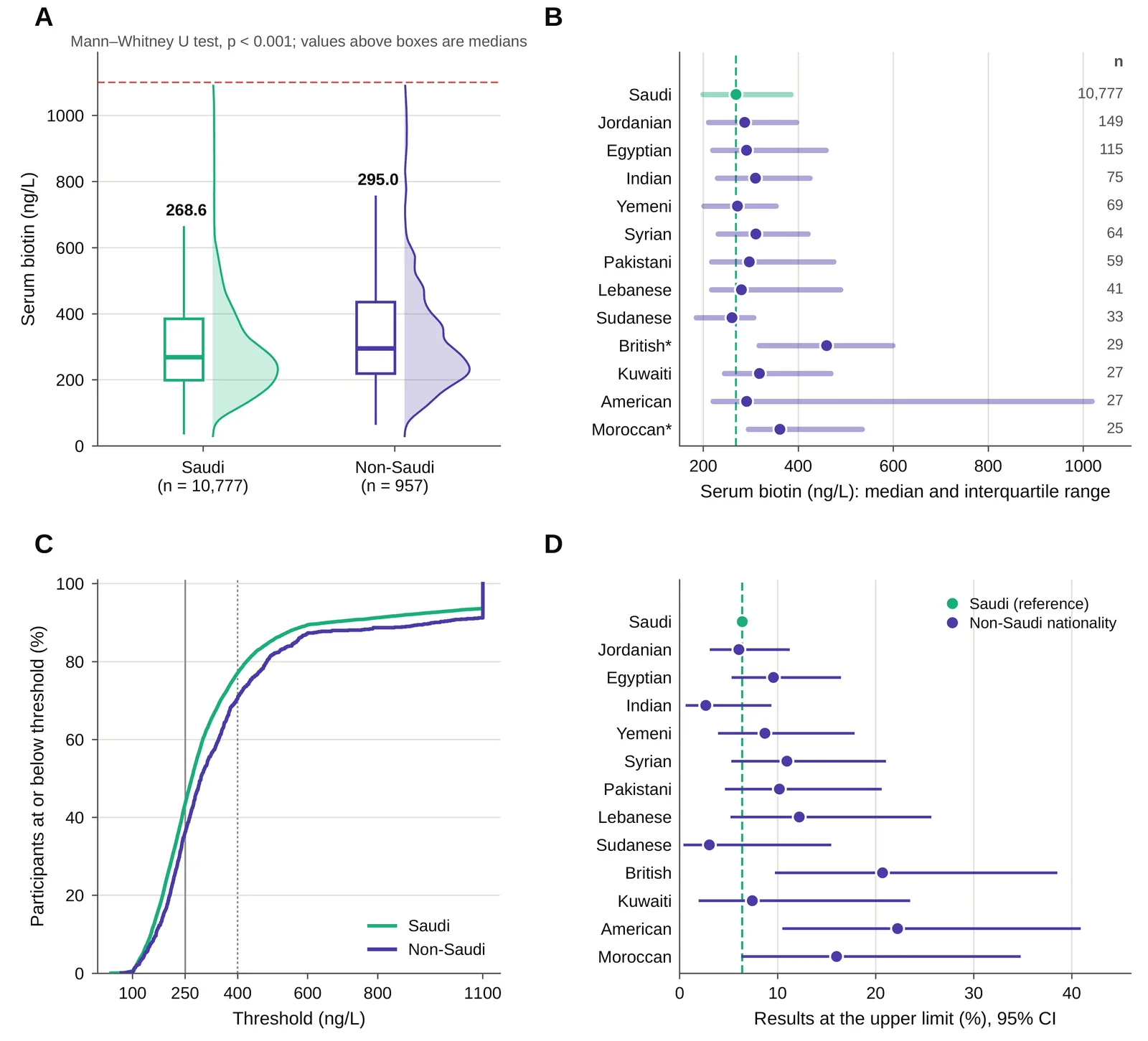
Serum biotin concentrations by nationality. (**A**) Box plots with density curves for Saudi and non-Saudi participants; medians (IQR width) were 268.6 (185.3) ng/L and 295.0 (216.1) ng/L, respectively (Mann–Whitney U test, *p* < 0.001); the dashed line marks the upper limit of the measuring range. (**B**) Median and interquartile range for Saudi participants and for each non-Saudi nationality with at least 25 participants, with the number of participants (*n*); the dashed line marks the Saudi median. (**C**) Cumulative distributions for Saudi and non-Saudi participants; vertical lines mark 250 ng/L (solid) and 400 ng/L (dotted). (**D**) Percentage of results at the upper limit of the measuring range by nationality, with 95% confidence intervals; the dashed line marks the Saudi percentage. * In panel (**B**), an asterisk marks the nationalities whose median differed from that of Saudi participants after Holm correction (*p* < 0.05). The distributions overlapped substantially.

As shown in Figure 5A, comparison of serum biotin levels between Saudi and non-Saudi participants revealed statistically significant differences (Mann–Whitney U test, *p* < 0.001). Saudi participants exhibited a slightly lower median biotin concentration (median = 268.6 ng/L; IQR = 185.3 ng/L) compared to non-Saudi participants (median = 295.0 ng/L; IQR = 216.1 ng/L). Box plot analysis demonstrated considerable overlap between groups, indicating that although the difference was statistically significant, its magnitude was modest and its interpretation is limited by the variables available in this dataset. Among the 12 non-Saudi nationalities with at least 25 participants (Figure 5B; Supplementary Table S1), medians ranged from 260.2 ng/L (Sudanese, *n* = 33) to 459.5 ng/L (British, *n* = 29); after Holm correction for multiple comparisons, only British (*p* < 0.001) and Moroccan (*n* = 25; *p* = 0.022) participants differed from Saudi participants. The remaining nationality categories each had fewer than 25 participants or were unspecified and were not compared individually. The cumulative distributions of Saudi and non-Saudi participants and the percentage of results at the upper limit by nationality are shown in Figure 5C,D.

### 3.7. Seasonal Variations in Serum Biotin Concentrations

**Figure 6:**
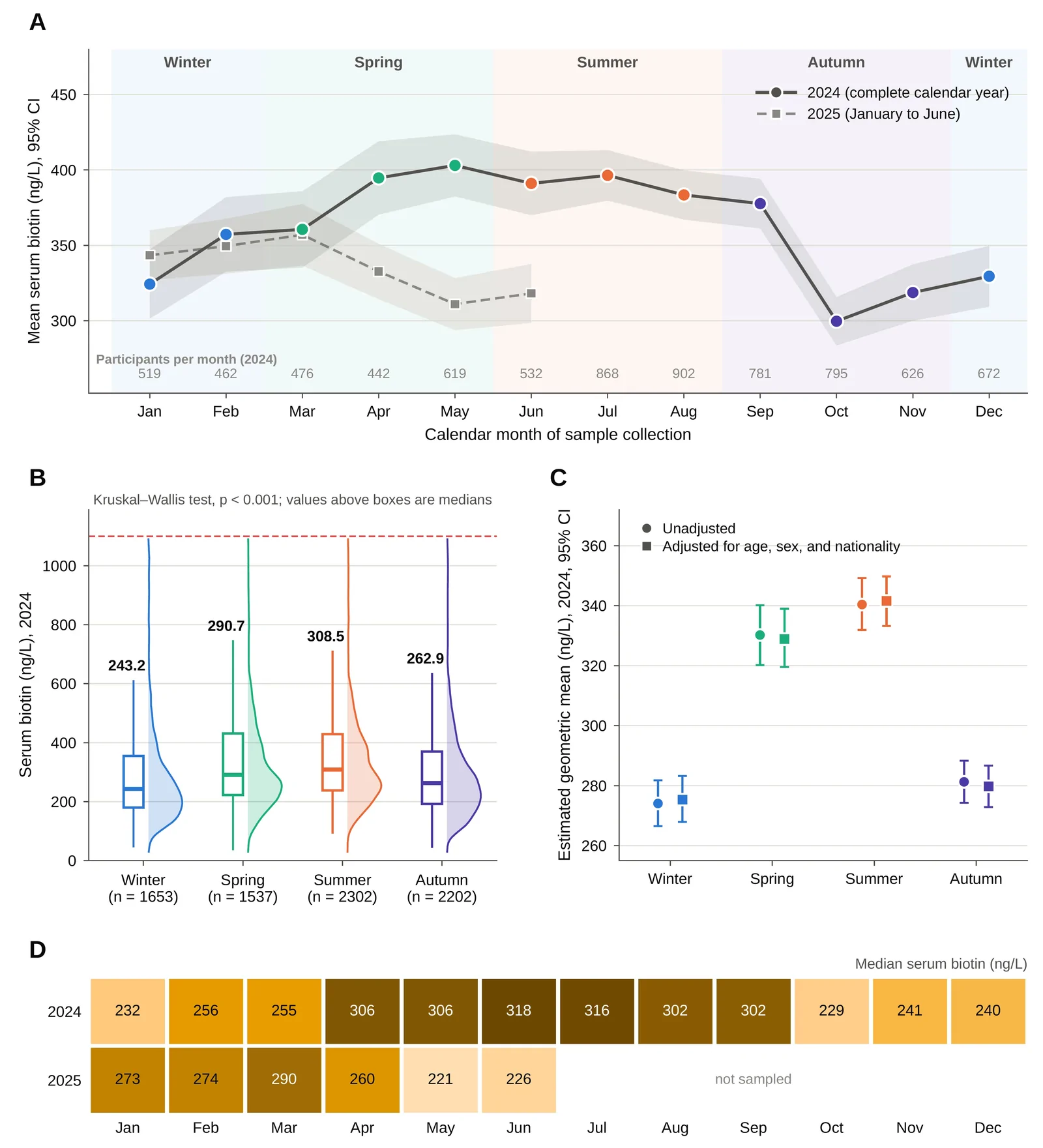
Seasonal variation in serum biotin concentrations. (**A**) Mean serum biotin concentration with 95% confidence interval by calendar month of sample collection in the complete calendar year 2024 (*n* = 7694; markers are colored by season, and numbers of participants are shown above the axis); the months January to June 2025 are shown for comparison (dashed line). (**B**) Box plots with density curves by meteorological season in 2024; medians were 243.2 ng/L in winter, 290.7 ng/L in spring, 308.5 ng/L in summer, and 262.9 ng/L in autumn (Kruskal–Wallis H = 277.0, *p* < 0.001). (**C**) Estimated geometric mean serum biotin by season in 2024 with 95% confidence interval from censored regression, unadjusted and adjusted for age, sex, and nationality. (**D**) Median serum biotin concentration by calendar month in 2024 and 2025. These differences should be interpreted as observational temporal patterns rather than evidence of causal seasonal effects (see also Supplementary Figures S1, S3 and S4).

Serum biotin concentrations exhibited a seasonal pattern in the complete calendar year 2024 (*n* = 7694; Kruskal–Wallis H = 277.0, *p* < 0.001). Monthly trends were characterized by a rise in B7 concentrations from winter to late spring and summer, followed by a decline in autumn (Figure 6A). Mean serum biotin levels rose from 324.3 ng/L in January to 394.7–403.0 ng/L in April and May and remained high through the summer (396.4 ng/L in July). Thereafter, concentrations declined in autumn (299.7 ng/L in October, the lowest monthly mean) before rising again by December (329.5 ng/L).

When stratified by season, the median serum biotin concentrations in 2024 were 243.2 ng/L in winter, 290.7 ng/L in spring, 308.5 ng/L in summer, and 262.9 ng/L in autumn. The Kruskal–Wallis analysis confirmed overall differences among the four seasons (H = 277.0, *p* < 0.001). As shown in Figure 6B, summer had the highest central tendency and winter the lowest. In censored regression adjusted for age, sex, and nationality, adjusted geometric means were higher in summer (341.6 ng/L, 95% CI: 333.2–349.8) and spring (328.8 ng/L, 95% CI: 319.5–339.0) than in autumn (279.7 ng/L, 95% CI: 272.8–286.7) and winter (275.3 ng/L, 95% CI: 267.9–283.2) (Figure 6C), and medians were higher in spring and summer than in winter and autumn in both sexes, in three age groups (18–34, 35–49, and ≥50 years), and in Saudi and non-Saudi participants (Supplementary Figure S3). However, the months January to June 2025 did not follow the 2024 pattern (for example, the June median was 317.9 ng/L in 2024 and 225.6 ng/L in 2025; Figure 6D), and concentrations changed in steps rather than gradually during the study period (Supplementary Figure S1; Supplementary Table S3), so a contribution of analytical (reagent-lot) variation cannot be excluded and the pattern requires confirmation over several years. Estimates from the pooled 18-month data are given in Supplementary Table S3 and Supplementary Figure S4. These findings describe cross-sectional temporal differences within the sampled cohort and should not be interpreted as evidence of causal biological seasonal effects.

### 3.8. Binary Logistic and Multivariable Linear Regression Analysis: Factors Associated with Serum Biotin Concentrations

To identify factors associated with a result at the upper limit of the measuring range, binary logistic regression was performed with a result at or above 1100 ng/L as the dependent variable. Three variables were included in the model: sex (female vs. male, reference: male), age (continuous, per 10 years), and nationality (non-Saudi vs. Saudi, reference: Saudi).

**Figure 7:**
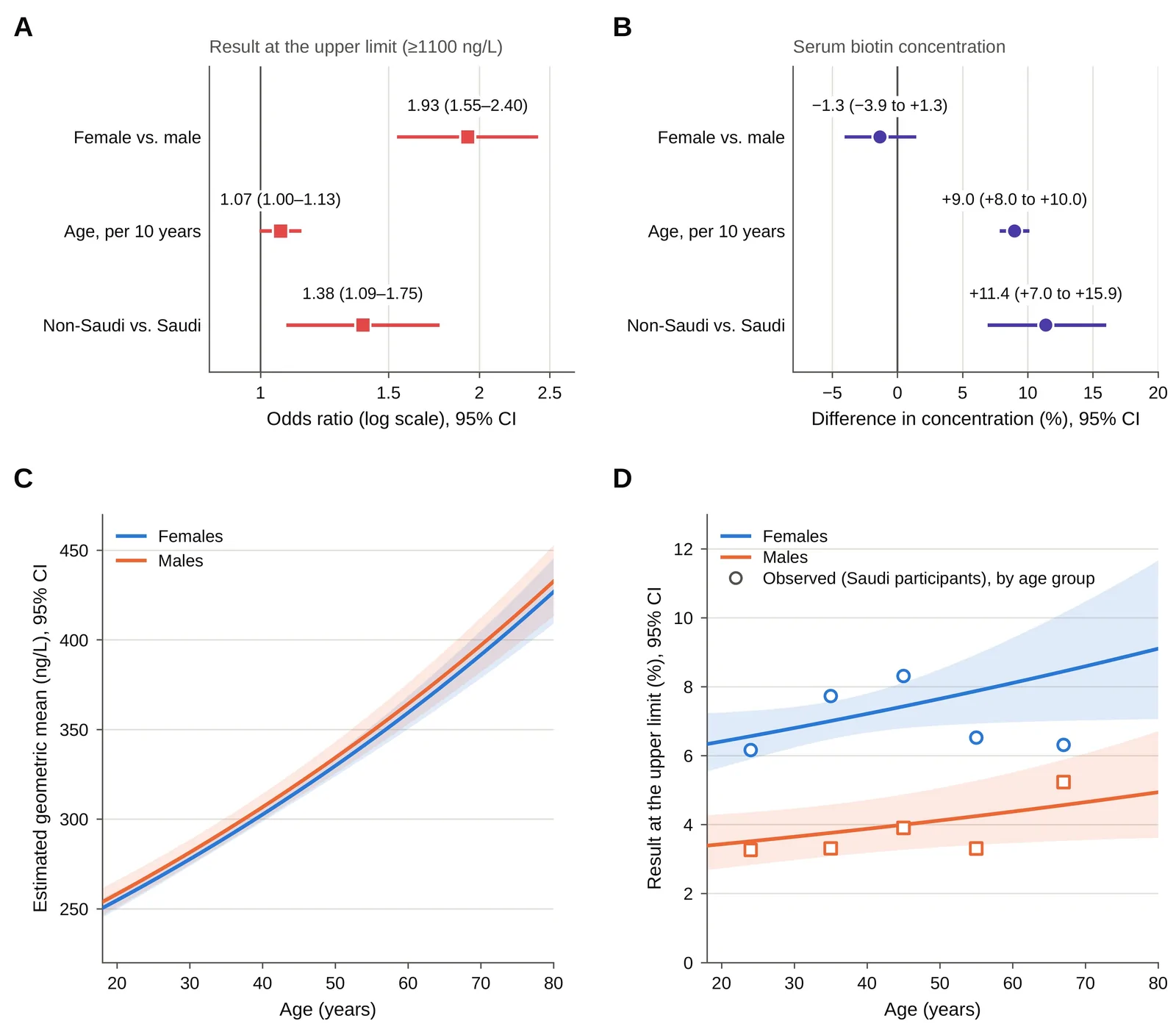
Regression analysis of factors associated with serum biotin concentrations. (**A**) Binary logistic regression model evaluating factors associated with a result at the upper limit of the measuring range (≥1100 ng/L); the effect measure is the odds ratio (log scale) with 95% confidence interval. (**B**) Censored linear regression of log-transformed serum biotin concentration; the effect measure is the percentage difference in concentration with 95% confidence interval. Panels (**A**,**B**) use different effect measures and are shown on separate axes. (**C**) Geometric mean serum biotin concentration by age in females and males estimated from the censored regression for Saudi participants, with 95% confidence bands. (**D**) Probability of a result at the upper limit by age in females and males estimated from the logistic regression for Saudi participants, with 95% confidence bands; open symbols show the observed percentages among Saudi participants by age group (18–29, 30–39, 40–49, 50–59, and ≥60 years). Both models included sex, age, and nationality (*n* = 11,733).

The model showed that sex, age, and nationality were associated with a result at the upper limit (Table 3 and Figure 7A). Female sex was associated with higher odds (β = 0.656, standard error [SE] = 0.111, z = 5.90, *p* < 0.001; OR = 1.93, 95% CI: 1.55–2.40), indicating that females had 93% higher odds compared to males after adjusting for age and nationality. Age demonstrated a weak positive association (β = 0.063, SE = 0.031, z = 2.03, *p* = 0.042; OR = 1.07 per 10 years, 95% CI: 1.00–1.13), which did not differ by sex (interaction *p* = 0.59) and departed from linearity (*p* < 0.001), without a monotonic trend across age groups (Figure 4D). Non-Saudi nationality was also associated with higher odds (β = 0.324, SE = 0.121, z = 2.68, *p* = 0.007; OR = 1.38, 95% CI: 1.09–1.75). Sex-specific estimates are given in Supplementary Table S4.

In the multivariable linear regression model, serum biotin concentration was analyzed as a continuous outcome to quantify the contribution of demographic factors. Variables included age (per 10 years), sex, and nationality (non-Saudi vs. Saudi, reference: Saudi). Because results at the upper limit are right-censored, the primary model was a censored regression of log-transformed concentration, with ordinary least-squares models of recorded values as sensitivity analyses (Table 4).

Age remained positively associated with serum biotin: concentrations were 9.0% higher per 10 years of age (95% CI: 8.0–10.0; *p* < 0.001), corresponding to 29.7 ng/L per 10 years (95% CI: 25.9–33.4) in the model of recorded values. The association was similar in females and males (8.9% and 9.1% per 10 years, respectively; interaction *p* = 0.82), showed no significant departure from linearity (restricted cubic spline, *p* = 0.86), and was similar when the analysis was restricted to ages 18–65 years (8.6% per 10 years).

Non-Saudi participants had 11.4% higher concentrations than Saudi participants (95% CI: 7.0–15.9; *p* < 0.001). Sex was not associated with concentration in the censored model (−1.3%, 95% CI: −3.9 to 1.3; *p* = 0.32), whereas in median regression, which is unaffected by censoring, concentrations were 7.6% lower in females (95% CI: −9.9 to −5.3; Supplementary Table S4). In the sensitivity analysis excluding results at the upper limit, concentrations were 19.7 ng/L lower in females (95% CI: −26.8 to −12.7), whereas the model of all recorded values showed no difference (8.2 ng/L, 95% CI: −1.7 to 18.2), reflecting the larger share of upper-limit results in females (Supplementary Figure S2B).

As illustrated in Figure 7, female sex showed the strongest association with a result at the upper limit of the measuring range (panel A, odds ratios), whereas age and nationality were associated with the concentration itself (panel B, percentage differences). Concentrations and the probability of an upper-limit result estimated from the two models by age and sex are shown in Figure 7C,D. A summary of the multivariable linear regression results is provided in Table 4. Under two illustrative models, the estimated concentrations of the 772 right-censored results had a median of about 1290 ng/L (IQR: 1180–1480; model A, log-normal upper tail) or about 1670 ng/L (IQR: 1330–2260; model B, continuation of the density observed below the limit), and the cohort mean would be about 371 or 407 ng/L instead of 351.9 ng/L, whereas the median would be unchanged. The log-normal model placed fewer results at or above the upper limit than were observed (1.6% vs. 6.6%); these estimates therefore depend strongly on the assumptions, are not measured values, and could be exceeded if the upper-limit results reflect supplement use (Supplementary Figure S5; Supplementary Table S5).

## 4. Discussion

This retrospective cross-sectional study provides large-scale (*n* = 11,735) descriptive epidemiological evidence on serum biotin distribution in an adult outpatient cohort in Riyadh, Saudi Arabia. Concentrations below 100 ng/L were uncommon, concentrations of 100–250 ng/L were frequent, and results at the upper limit of the assay were observed in a minority of participants. Serum biotin concentrations varied by age, sex, nationality, and season; these associations should be considered descriptive because the dataset was derived from an outpatient clinical/laboratory testing setting and did not include detailed clinical, renal, dietary, behavioral, or supplementation data. Because the cohort was drawn from clinically indicated outpatient testing rather than probability-based population sampling, the observed category frequencies do not necessarily reflect the national prevalence estimates. The cohort was predominantly female (79.0%) and Saudi (91.8%) and was drawn from a single private medical group in Riyadh; estimates for males and for non-Saudi residents are therefore less precise, and the findings may not be generalizable to other regions, to people who do not use private outpatient services, or to the general population. Because the reasons for requesting the test were not recorded, people with symptoms attributed to biotin deficiency and people already taking biotin may both be over-represented, and the direction of the resulting selection bias is unknown.

The median serum biotin concentration of 270.9 ng/L is lower than the range of 400–1200 ng/L quoted for blood biotin in a European clinical series [19], though direct comparisons are limited by methodological variations and a lack of universally standardized cutoff values [10]. Symptomatic disorders of biotin metabolism presenting in adulthood are rare [20]. The substantial proportion of concentrations at or below 250 ng/L (42.9%) warrants consideration, although it is threshold-dependent (76.6% of results were below 400 ng/L) and does not indicate functional deficiency; marginal deficiency may have clinical relevance during periods of increased metabolic demand such as pregnancy, where it is common and may have teratogenic implications [8,12,21]. However, clinical manifestations typically occur only at substantially lower concentrations [22,23], and the clinical significance of the 100–250 ng/L range requires investigation through longitudinal studies examining metabolic biomarkers and clinical outcomes.

Biotin is widely distributed in foods and can also be synthesized by intestinal microbiota [24,25], which may contribute generally to the relative rarity of overt deficiency in adults. Recognized deficiency is more often described in higher-risk settings, including inflammatory bowel disease, chronic alcoholism, and genetic defects in biotin metabolism [5,25]. Although concentrations below 100 ng/L were rare in our cohort (0.5%), this subgroup remains clinically important. Given the small number of such individuals, detailed subgroup analyses were not feasible and were therefore not performed. Low concentrations may be associated with inadequate dietary intake, malabsorption, chronic illness, or medication use; however, the absence of detailed clinical, dietary, supplementation, and renal data limits further characterization of this group. In addition, subclinical or undocumented conditions affecting nutrient absorption could not be fully excluded. Results at the upper limit of the measuring range were observed in 6.6% of participants; because post-dilution values were not available, their true concentrations are unknown. In other settings, such values may reflect supplementation practices, particularly given the popularity of biotin-containing products marketed for hair, skin, and nail benefits [19,26,27]; in one clinical series, all women who were already taking 5 mg of oral biotin had serum concentrations above 1000 ng/L [19]. However, supplementation history was unavailable in our cohort, so this explanation could not be directly evaluated. Importantly, high biotin exposure may have clinically significant implications because of interference with laboratory immunoassays, potentially leading to falsely elevated or decreased results in cardiac troponin, thyroid function tests, and hormone assays [28,29]. These findings highlight the need for targeted evaluation of at-risk populations and for future studies incorporating clinical, nutritional, and metabolic assessments.

Females exhibited significantly higher odds of a result at the upper limit of the assay (OR = 1.93, 95% CI: 1.55–2.40, *p* < 0.001; 7.3% vs. 3.9%), though males showed slightly higher median concentrations (287.9 ng/L vs. 266.8 ng/L). These findings are compatible: within the measuring range, concentrations were lower in females (19.7 ng/L lower after exclusion of results at the upper limit), whereas a larger share of females had results at the upper limit, so that mean recorded concentrations and the censored regression estimate did not differ by sex. In other populations, women often report greater use of dietary supplements, including products marketed for cosmetic benefits [30,31]; however, supplementation data were not available in our cohort, so this possible explanation could not be evaluated directly. The modest difference in median concentrations, together with the non-significant sex effect in ANOVA, suggests that sex-related differences in overall circulating biotin levels are limited within the central range of the distribution, whereas differences may become more apparent at the upper extreme.

Serum biotin showed a weak but statistically significant positive association with age (Spearman ρ = 0.18, *p* < 0.001), with mean concentrations increasing through the 66–75-year age group and no clear change thereafter. Estimates in the oldest strata should be interpreted cautiously because the number of participants aged ≥ 76 years was small. Age-related differences in supplement use and nutritional patterns have been reported in other populations [32,33], and physiological changes with aging may also influence nutrient status [34]; however, the present dataset did not include supplementation history, renal function, or detailed clinical covariates, so the mechanisms underlying the observed age-related pattern cannot be determined. Younger adults (18–39 years) were more frequently represented in the 100–250 ng/L band, which may be relevant from a public health perspective, particularly for women of childbearing age, in whom marginal biotin deficiency has been linked to adverse fetal effects [8,35]. The overrepresentation of older adults (≥50 years) in the >250 to <1100 ng/L band should therefore be interpreted as a descriptive pattern rather than evidence of more adequate nutritional practices, although it reinforces the importance of obtaining supplement history when interpreting biotin results in routine care [36].

We observed a statistically significant seasonal variation in serum biotin levels, with a summer peak (median 308.5 ng/L) and lower values in winter and autumn (medians 243.2 and 262.9 ng/L; Kruskal–Wallis H = 277.0, *p* < 0.001) in the complete calendar year 2024. Higher values in spring and summer than in winter and autumn persisted after adjustment for age, sex, and nationality and were seen in every demographic subgroup examined, but the pattern was not reproduced in the first half of 2025. This seasonal pattern has not been widely described for serum biotin and may reflect temporal variation in dietary intake, supplement exposure, unmeasured patient characteristics such as the indication for testing, analytical (reagent-lot) variation, or other unmeasured factors. Seasonal differences in dietary intake and eating patterns [37,38] and seasonal variation in gut microbiome composition [39,40] have been reported in other populations; however, these factors were not measured in the present dataset. Therefore, the observed seasonal contrast should be interpreted as a descriptive temporal pattern and as a basis for hypothesis generation in future prospective studies.

Saudi participants exhibited slightly lower median biotin concentrations compared to non-Saudi participants. This difference persisted in multivariable analysis (11.4% higher concentrations in non-Saudi participants). Among individual nationalities with at least 25 participants, only British and Moroccan participants differed from Saudi participants after correction for multiple comparisons, and these subgroups were small (*n* = 29 and *n* = 25). In principle, nationality may capture heterogeneous dietary, cultural, or genetic backgrounds relevant to biotin metabolism [5,8,41,42]; however, the present dataset does not permit mechanistic interpretation because detailed dietary, supplementation, and clinical data were unavailable and several non-Saudi subgroups were small. Accordingly, the observed nationality differences should be interpreted as modest demographic associations rather than as evidence that ethnicity or nationality is itself a major determinant of biotin status.

The binary logistic regression analysis identified female sex (OR = 1.93, 95% CI: 1.55–2.40) and non-Saudi nationality (OR = 1.38, 95% CI: 1.09–1.75) as factors associated with a result at the upper limit of the assay, whereas the association with age was weak and not monotonic (OR = 1.07 per 10 years, 95% CI: 1.00–1.13). Because this outcome is defined by the measuring range of the assay and may reflect supplement use, which was not recorded, these associations should not be interpreted as determinants of biotin status. The relatively modest odds ratios indicate that while these factors are statistically significant in a large sample, their individual predictive power for identifying individuals with results at the upper limit is limited, suggesting that clinical prediction of biotin status requires direct measurement rather than reliance on demographic characteristics.

Our findings have several potential clinical and public health implications, but these should be interpreted conservatively. The substantial proportion of concentrations at or below 250 ng/L (42.9%) supports further evaluation of whether this range has functional significance, including through biomarkers such as urinary 3-hydroxyisovaleric acid or lymphocyte propionyl-CoA carboxylase activity [18]. General dietary sources of biotin, including eggs, nuts, seeds, legumes, and whole grains, remain relevant in discussions of nutritional adequacy [42], but the present data do not justify intervention-oriented conclusions regarding dietary counseling or supplementation policy. The observed proportion of results at the upper limit of the assay highlights the importance of patient and provider awareness regarding biotin-containing products, especially given the limited evidence supporting cosmetic supplementation in biotin-sufficient individuals [17] and the growing concern that over-the-counter biotin use may interfere with laboratory testing and contribute to diagnostic error [42,43,44].

The identification of younger adults as more likely to have concentrations of 100–250 ng/L may justify closer attention in future targeted studies, particularly among women of childbearing age given evidence that marginal biotin deficiency during pregnancy may have adverse fetal effects [8,35]. However, this cross-sectional dataset does not support firm recommendations regarding targeted screening, population-wide fortification, or specific intervention strategies. Questions regarding high-risk groups, including pregnant women and individuals with malabsorptive disorders or long-term anticonvulsant use, require dedicated prospective evaluation [6].

Taken together, our findings provide descriptive information on serum biotin concentrations in a large outpatient clinical/laboratory cohort and identify several priorities for future prospective research. Clarifying the clinical relevance of low and over-range biotin concentrations will require studies that combine serum testing with supplementation history, renal function, detailed dietary assessment, and functional biomarkers of biotin status.

### Limitations

This study has several important limitations. First, its retrospective cross-sectional design precludes causal inference and captures only a single time point per participant. Second, the cohort was derived from an outpatient clinical/laboratory testing setting rather than population-based sampling, introducing heterogeneity in clinical indications and healthcare utilization that may limit generalizability beyond similar institutional clinical settings; the reasons for requesting the test were not recorded, so selection related to symptoms or to supplement use cannot be quantified. Third, key potential confounders were unavailable, including dietary intake, supplementation history, pregnancy status, renal function, smoking, alcohol use, inflammatory bowel disease, other malabsorptive conditions, medication history, and broader comorbidity burden; residual confounding is therefore likely. In particular, the higher odds of an upper-limit result in females and the 6.6% of results at the upper limit may largely reflect the indication for testing and supplement use. Fourth, results above the measuring range were recorded at its upper limit, and post-dilution values were not available; these right-censored results (6.6%) lead to underestimation of the mean, and the ≥1100 ng/L band cannot be interpreted as a defined high status. Fifth, plate and kit-lot identifiers were not available and July to December were sampled in a single calendar year, so analytical variation cannot be separated from seasonal variation. Sixth, serum biotin was the only biomarker assessed. Although analytically robust, serum concentration may not fully reflect tissue status or metabolic adequacy, and functional biomarkers such as urinary 3-hydroxyisovaleric acid, 3-hydroxyisovaleryl carnitine, or lymphocyte propionyl-CoA carboxylase activity were not available. Seventh, the oldest age strata were small, and the cohort was predominantly female and Saudi, which further limits subgroup inference and external validity. Finally, the concentration bands are derived from the underlying continuous biotin measure and rest on thresholds that are not standardized; band frequencies are therefore threshold-dependent and should not be interpreted as the prevalence of deficient or adequate status [45,46,47].

## 5. Conclusions

This study provides a comprehensive characterization of serum biotin concentrations in a large Saudi Arabian outpatient clinical/laboratory cohort. While concentrations below 100 ng/L were rare, concentrations of 100–250 ng/L were frequent and results at the upper limit of the assay were observed in a notable minority. Age, sex, nationality, and season showed statistical associations with serum biotin concentrations, but these findings should be interpreted cautiously because of the retrospective cross-sectional design, cohort heterogeneity, and lack of detailed clinical, renal, dietary, and supplementation data. Our findings provide descriptive information and support prospective studies incorporating functional biomarkers and richer covariate data to clarify the clinical significance of low and over-range biotin concentrations.

## Figures and Tables

**Table 1:** Cohort characteristics and serum biotin summary (*n* = 11,735).

Section	Variable/Category	Count (*N*)	Statistics (%)	Mean ± SD	Median (IQR)/Range
A. Cohort Overview	Total participants	11,735	–	–	–
	Age (years)	–	–	36.5 ± 11.8	Median = 34 (28–42); Range = 18–100
	Serum biotin (ng/L)	–	–	351.9 ± 251.8	Median = 270.9 (200.8–388.3); Range = 38.3–1176.3
B. Age Summary	Count	11,735	–	–	–
	Mean	–	–	36.53	–
	Std (SD)	–	–	11.76	–
	Min	–	–	18.00	–
	25th percentile	–	–	28.00	–
	50th percentile (Median)	–	–	34.00	–
	75th percentile	–	–	42.00	–
	Max	–	–	100.00	–
C. Demographic Composition	Female	9270	79.0%	–	–
	Male	2464	21.0%	–	–
	Sex not recorded	1	<0.1%	–	–
	Saudi nationals	10,778	91.8%	–	–
	Non-Saudi nationals	957	8.2%	–	–
D. Serum biotin concentration band *	<100 ng/L	62	0.5%	80.73 ± 14.53	Median = 85.57
	100–250 ng/L	4977	42.4%	187.43 ± 40.32	Median = 191.72
	>250 to <1100 ng/L	5923	50.5%	395.48 ± 160.91	Median = 345.08
	≥1100 ng/L †	772	6.6%	1100.18 ± 3.49	Median = 1100.00
	All results <1100 ng/L (sensitivity analysis)	10,962	93.4%	299.24 ± 160.25	Median = 261.04

Serum biotin is reported in ng/L (11,734 results; one participant had no numeric result); SD = standard deviation; IQR = interquartile range. * Bands are descriptive concentration ranges and do not denote clinical nutritional status. † Results above the measuring range were recorded at its upper limit (1100 ng/L) and are right-censored; means and SDs that include them underestimate the true values.

**Table 2:** Serum biotin concentration bands by sex and age group.

Group	*n*	<100 ng/L, *n* (%)	100–250 ng/L, *n* (%)	>250 to <1100 ng/L, *n* (%)	≥1100 ng/L, *n* (%)	Median (IQR), ng/L
All participants	11,734	62 (0.5)	4977 (42.4)	5923 (50.5)	772 (6.6)	270.9 (200.8–388.3)
Female	9269	59 (0.6)	4037 (43.6)	4498 (48.5)	675 (7.3)	266.8 (198.0–381.8)
Male	2464	3 (0.1)	939 (38.1)	1425 (57.8)	97 (3.9)	287.9 (212.4–406.3)
18–29 years	3525	24 (0.7)	1761 (50.0)	1539 (43.7)	201 (5.7)	248.1 (188.1–333.2)
30–39 years	4518	30 (0.7)	2005 (44.4)	2173 (48.1)	310 (6.9)	262.5 (197.8–376.5)
40–49 years	2057	5 (0.2)	800 (38.9)	1090 (53.0)	162 (7.9)	284.2 (211.4–409.2)
50–59 years	948	1 (0.1)	267 (28.2)	623 (65.7)	57 (6.0)	314.3 (240.3–459.7)
≥60 years	686	2 (0.3)	144 (21.0)	498 (72.6)	42 (6.1)	382.6 (266.1–531.0)

Values are numbers (row percentages) of participants with a numeric result in each concentration band. The bands are derived from the underlying serum biotin concentration and are presented descriptively; concentrations were not compared statistically across bands. The ≥1100 ng/L band contains the results recorded at the upper limit of the measuring range. IQR = interquartile range.

**Table 3:** Factors associated with a serum biotin result at the upper limit of the measuring range (≥1100 ng/L): binary logistic regression (*n* = 11,733; 772 events).

Variable	Coefficient (β)	Std. Error	z-Value	*p*-Value	Odds Ratio (95% CI)
Sex (female vs. male)	0.656	0.111	5.90	<0.001	1.93 (1.55–2.40)
Age (per 10 years)	0.063	0.031	2.03	0.042	1.07 (1.00–1.13)
Nationality (non-Saudi vs. Saudi)	0.324	0.121	2.68	0.007	1.38 (1.09–1.75)

**Table 4:** Multivariable linear regression for serum biotin concentration (*n* = 11,733).

Predictor	Censored Regression: Difference, % (95% CI)	*p*	All Recorded Values: β, ng/L (95% CI)	*p*	Excluding Results at Upper Limit: β, ng/L (95% CI)	*p*
Age (per 10 years)	9.0 (8.0 to 10.0)	<0.001	29.7 (25.9 to 33.4)	<0.001	28.2 (25.4 to 31.0)	<0.001
Sex (female vs. male)	−1.3 (−3.9 to 1.3)	0.32	8.2 (−1.7 to 18.2)	0.11	−19.7 (−26.8 to −12.7)	<0.001
Nationality (non-Saudi vs. Saudi)	11.4 (7.0 to 15.9)	<0.001	38.4 (20.6 to 56.2)	<0.001	22.7 (11.4 to 34.0)	<0.001

All models included age, sex, and nationality; male sex and Saudi nationality were the reference categories. Censored regression: Tobit model of log-transformed concentration with right-censoring at 1100 ng/L; coefficients are expressed as percentage differences. All recorded values and the analysis excluding results at the upper limit (*n* = 10,961): ordinary least-squares models with robust standard errors; β is the difference in ng/L. CI = confidence interval.

## Data Availability

The datasets analyzed during the current study are not publicly available because they comprise anonymized clinical and laboratory records governed by institutional data protection requirements. De-identified data may be available from the corresponding authors on reasonable request, subject to approval by Dr. Sulaiman Al Habib Medical Group and applicable institutional and ethical restrictions.

## Supplementary Materials

The following supporting information can be downloaded at: https://www.mdpi.com/article/10.3390/biomedicines14102320/s1, Figure S1: Serum biotin over the study period and data-derived analytical periods; Figure S2: Serum biotin by age and sex; Figure S3: Seasonal pattern in 2024 by sex, age group, and nationality; Figure S4: Seasonal estimates in the pooled 18-month data under four adjustment specifications; Figure S5: Model-based estimates of the results recorded at the upper limit of the measuring range; Table S1: Participants and serum biotin by nationality; Table S2: Serum biotin by age group and sex; Table S3: Temporal analyses; Table S4: Regression models, including sex-specific, nonlinearity, and sensitivity analyses; Table S5: Model-based estimates for each result recorded at the upper limit (Excel file).

## Abbreviations

The following abbreviations are used in this manuscript:

4-PL	four-parameter logistic
ANOVA	analysis of variance
CI	confidence interval
CV	coefficient of variation
ELISA	enzyme-linked immunosorbent assay
IQR	interquartile range
IRB	Institutional Review Board
KDE	kernel density estimation
LoB	limit of blank
LoD	limit of detection
LoQ	limit of quantitation
LOWESS	locally weighted scatterplot smoothing
OR	odds ratio
SD	standard deviation
SE	standard error
