# Supplementary material for: Determinants of Serum Biotin (Vitamin B7) Concentrations: Influence of Age, Sex, and Season in a Large Population Cohort: A Retrospective Cross-Sectional Study
Source: Biomedicines. 2026 Oct 13;14(10):2320. doi: 10.3390/biomedicines14102320 (PMC13701354; doi:10.3390/biomedicines14102320)
Supplement: Supplementary file 1 [file biomedicines-14-02320-s001.zip › Supplementary_Materials.pdf]

Supplementary Materials

Determinants of Serum Biotin (Vitamin B7) Concentrations: Influence of Age, Sex, and Season in a Large Population Cohort: a retrospective cross-sectional study

Contents. Figure S1: Serum biotin over the study period and data-derived analytical periods. Figure S2: Serum biotin by age and sex. Figure S3: Seasonal pattern in 2024 by sex, age group, and nationality. Figure S4: Seasonal estimates in the pooled 18-month data under four adjustment specifications. Figure S5: Model-based estimates of the results recorded at the upper limit of the measuring range. Table S1: Participants and serum biotin by nationality. Table S2: Serum biotin by age group and sex. Table S3: Temporal analyses. Table S4: Regression models, including sex-specific, nonlinearity, and sensitivity analyses. Table S5: Model-based estimates for each result recorded at the upper limit (Excel file; summary below).

All analyses are based on the 11,734 participants with a numeric serum biotin result. Results above the measuring range of the assay were recorded at its upper limit (1,100 ng/L) and are treated as right-censored.

Supplementary Figures

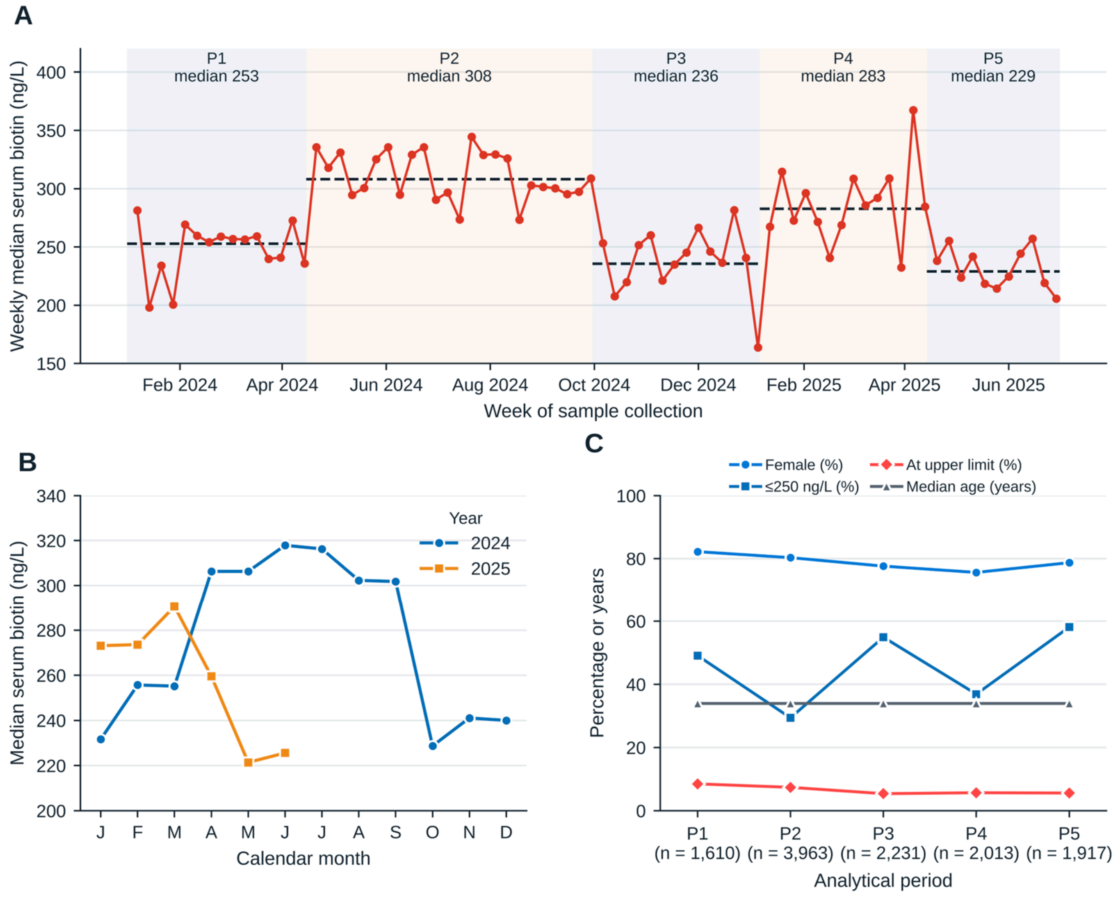

Figure S1. Serum biotin over the study period and data-derived analytical periods. (A) Median serum biotin concentration by week of sample collection. Shaded bands mark five periods (P1–P5) identified by change-point analysis of the weekly median log-transformed concentration (number of periods chosen by the Bayesian information criterion); dashed lines show the median of each period. (B) Median serum biotin

concentration by calendar month, shown separately for 2024 and 2025; the months January to June were sampled in both years. (C) Characteristics of the participants tested in each period: percentage female, median age, percentage with a concentration at or below 250 ng/L, and percentage with a result at the upper limit of the measuring range. The periods were derived from the data and are not recorded reagent lots or calibration events; plate and kit-lot identifiers were not available.

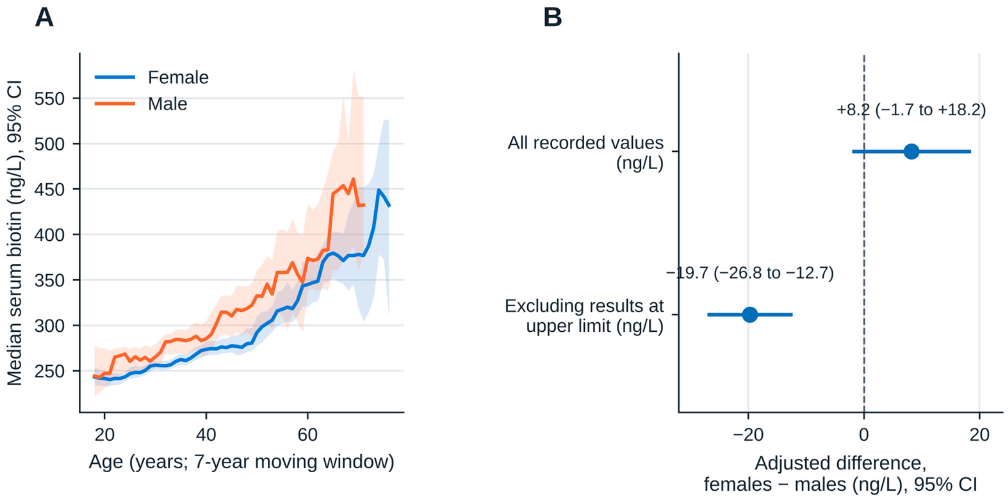

Figure S2. Serum biotin by age and sex. (A) Median serum biotin concentration with 95% confidence interval by age in females and males (7-year moving window; ages with fewer than 40 participants in the window are not shown). (B) Adjusted difference in serum biotin concentration between females and males (females − males, ng/L) with 95% confidence interval, from ordinary least-squares models adjusted for age and nationality, for all recorded values and after exclusion of results at the upper limit of the measuring range.

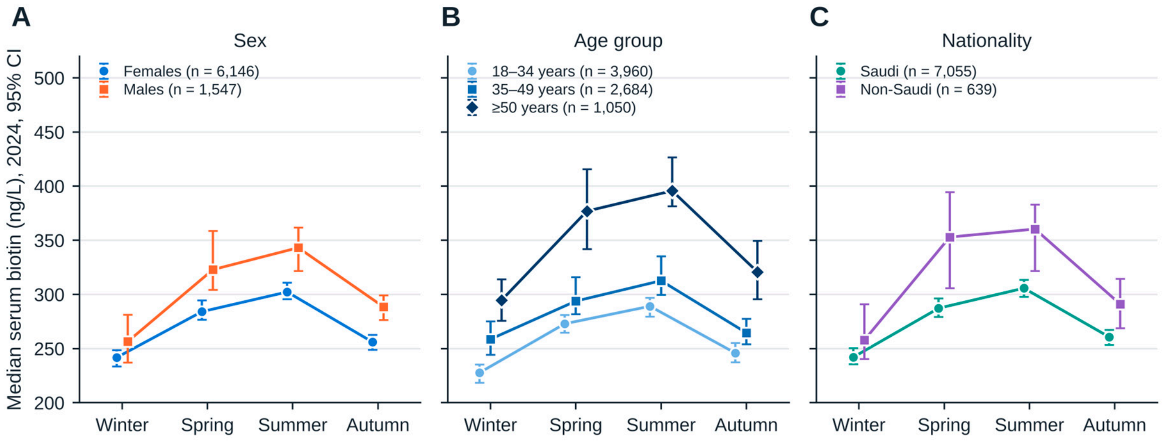

Figure S3. Seasonal pattern of serum biotin in the complete calendar year 2024 by (A) sex, (B) age group, and (C) nationality. Points are medians with 95% confidence intervals. Medians were higher in spring and summer than in winter and autumn in every subgroup.

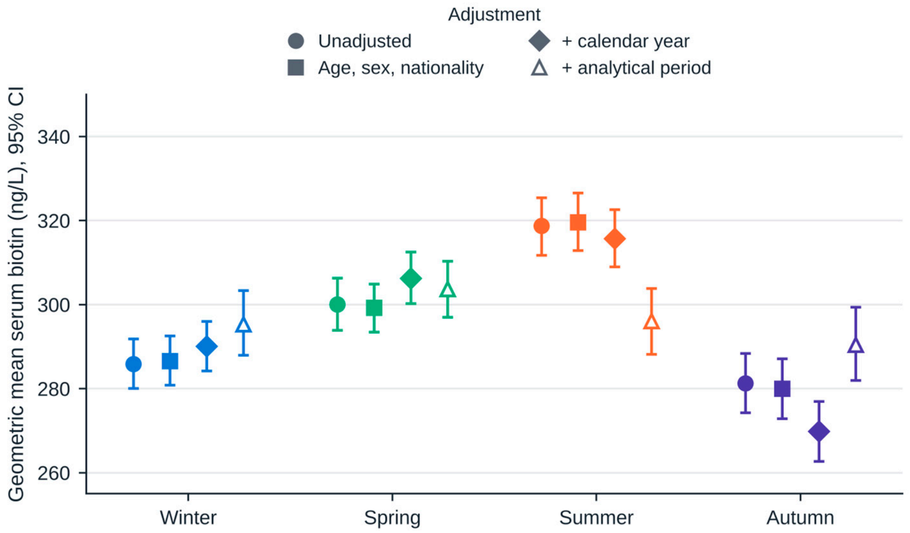

Figure S4. Geometric mean serum biotin concentration by season in the pooled 18-month data, with 95% confidence interval, from censored (Tobit) regression under four specifications: unadjusted; adjusted for age, sex, and nationality; additionally adjusted for calendar year; and adjusted for age, sex, nationality, and data-derived analytical period in place of calendar year (Figure S1). Estimates are standardized to the covariate distribution of the whole cohort.

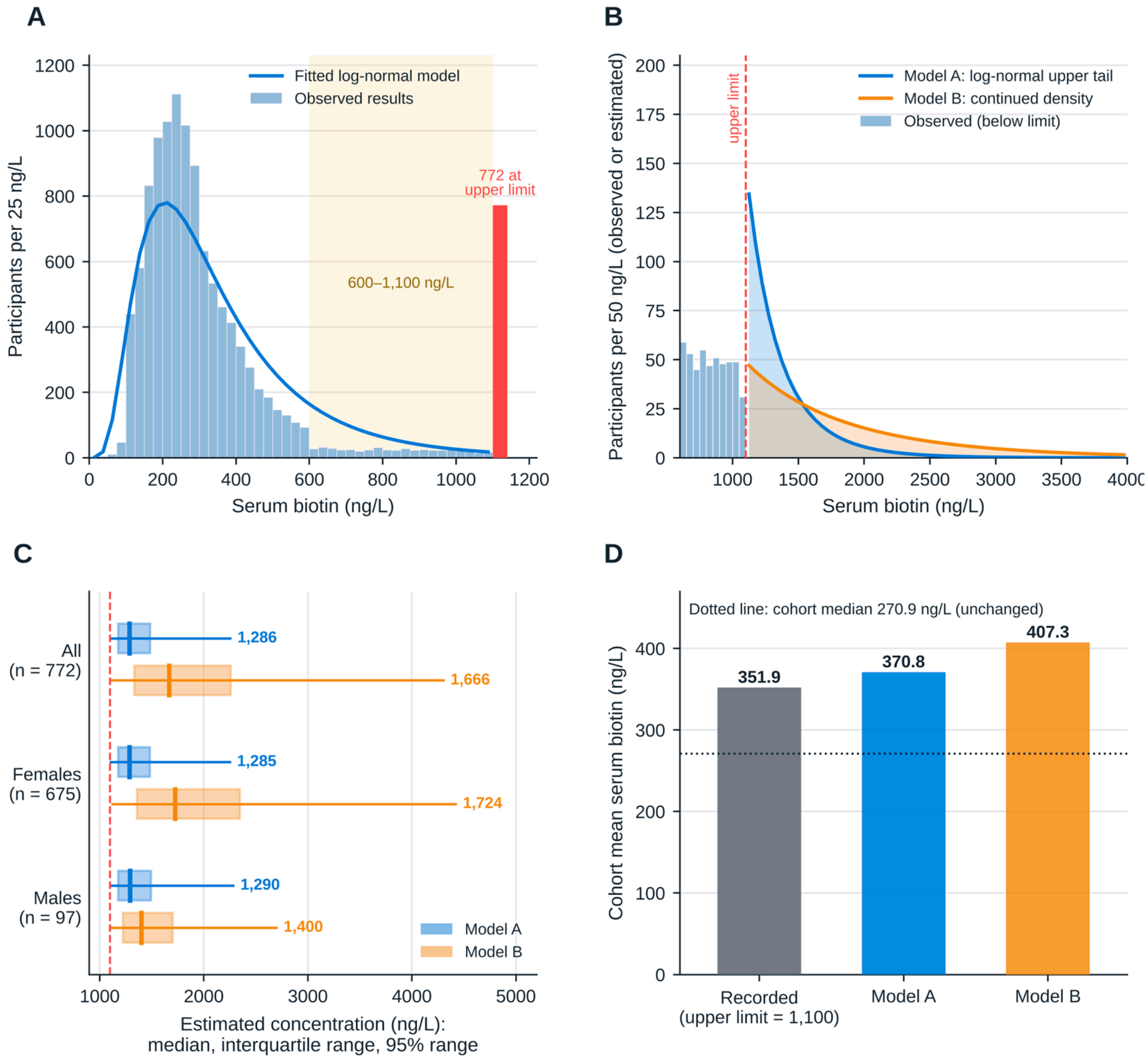

Figure S5. Model-based estimates of the results recorded at the upper limit of the measuring range (n = 772). (A) Distribution of the results below the upper limit (bars), the fitted censored log-normal regression (line), and the results recorded at the upper limit (red bar). The model does not reproduce the nearly flat density between 600 and 1,100 ng/L (shaded). (B) Observed results between 600 and 1,100 ng/L and the estimated distribution of the right-censored results above the limit under model A (upper tail of the log-normal model, conditional on a value at or above the limit) and model B (the density observed between 600 and 1,100 ng/L continued above the limit as an exponential tail, by sex). (C) Median, interquartile range, and 95% range of the estimated concentrations, overall and by sex. (D) Cohort mean with the upper-limit results taken as recorded and under each model; the dotted line shows the cohort median, which is not affected. The estimates are model-based and are not measured concentrations.

Supplementary Tables

Table S1. Participants and serum biotin by nationality (n = 11,734).

Nationality	n	Median (IQR), ng/L	At upper limit, %	p vs. Saudi*
Saudi (reference)	10,777	268.6 (199.2–384.5)	6.4	—
Jordanian	149	286.9 (210.8–396.3)	6.0	0.77
Egyptian	115	290.8 (219.7–458.6)	9.6	0.50
Indian	75	309.7 (229.6–424.6)	2.7	0.50
Yemeni	69	271.7 (201.1–353.4)	8.7	0.77
Syrian	64	310.4 (231.3–420.6)	10.9	0.50
Pakistani	59	296.7 (217.4–474.6)	10.2	0.50
Lebanese	41	280.1 (217.3–489.2)	12.2	0.77
Sudanese	33	260.2 (185.2–306.0)	3.0	0.77
British	29	459.5 (317.4–599.2)	20.7	<0.001
Kuwaiti	27	318.0 (244.5–468.6)	7.4	0.50
American	27	291.0 (220.5–1018.5)	22.2	0.50
Moroccan	25	361.1 (294.7–534.6)	16.0	0.022

* Mann–Whitney U test against Saudi participants, adjusted for 12 comparisons by the Holm method. IQR = interquartile range. Upper limit = result recorded at the upper limit of the measuring range (≥1,100 ng/L). A further 36 participants had a nationality recorded as "other" or with a code that could not be assigned to a country; they are included among non-Saudi participants.

Table S1 (continued). Nationalities with fewer than 25 participants (69 nationalities; 208 participants). These groups were not compared individually.

Participants	Nationality	Total n
n = 23	Palestinian	23
n = 21	Canadian	21
n = 12	Australian	12
n = 10 each	Filipino, Ukrainian	20
n = 8	Bahraini	8
n = 7	German	7
n = 6 each	French, Serbian	12
n = 5 each	Russian, Tunisian	10
n = 4 each	Brazilian, Chinese, Senegalese, South African	16
n = 3 each	Azeri, Belarusian, Iraqi, Sahrawi, Sierra Leonean, Sri Lankan	18
n = 2 each	Afghan, Algerian, Chadian, Colombian, Eritrean, Ethiopian, Greek, Korean, Malian, Mexican, Seychellois, Singaporean, Swedish	26
n = 1 each	Andorran, Angolan, Austrian, Belgian, Bengali, Bosnian and Herzegovinian, Chilean, Czech, Dutch, Emirati, Gambian, Georgian, Hungarian, Iranian, Irish, Japanese, Kazakhstani, Laotian, Lithuanian, Macedonian, Malagasy, Mauritanian, Mauritian, Nepalese, Omani, Peruvian, Polish, Qatari, Romanian, Scottish, Slovak, Spanish, St. Kitts and Nevis, Turkish, Venezuelan	35

Table S2. Serum biotin by age group and sex.

Age, years	Females, n	Mean ± SD, ng/L	Median, ng/L	Upper limit, %	Males, n	Mean ± SD, ng/L	Median, ng/L	Upper limit, %
18–25	1,340	301.1 ± 222.0	240.6	4.6	341	309.7 ± 208.2	255.0	3.8
26–35	3,946	341.2 ± 261.7	255.3	7.7	894	325.0 ± 199.1	276.5	3.6
36–45	2,283	367.7 ± 272.1	272.7	8.3	728	352.6 ± 224.4	290.2	4.4
46–55	944	378.7 ± 259.7	291.6	7.6	238	371.0 ± 209.0	333.2	2.9
56–65	568	413.8 ± 241.4	348.4	5.6	161	409.4 ± 230.6	367.6	3.1
66–75	160	462.6 ± 272.5	385.0	8.8	67	529.4 ± 285.3	453.7	9.0
76–85	25	530.6 ± 314.4	482.0	8.0	31	463.4 ± 272.5	431.1	6.5
≥86	3	400.0 ± 27.3	400.5	0.0	4	540.7 ± 329.6	413.9	0.0

Upper limit, % = percentage of results recorded at the upper limit of the measuring range (≥1,100 ng/L). Means and standard deviations include results recorded at the upper limit of the measuring range (1,100 ng/L) and therefore underestimate the true values. One participant without a recorded sex is not shown.

Table S3. Temporal analyses.

A. Data-derived analytical periods

Period	Dates	n	Median (IQR), ng/L	≤250 ng/L, %	Upper limit, %	Female, %	Age, median	Non-Saudi, %
P1	1 Jan 2024 – 14 Apr 2024	1,610	252.7 (188.9–371.7)	49.1	8.5	82.2	34	9.9
P2	15 Apr 2024 – 29 Sep 2024	3,963	308.1 (239.3–431.6)	29.4	7.4	80.3	34	7.1
P3	30 Sep 2024 – 5 Jan 2025	2,231	235.7 (166.9–338.8)	54.9	5.4	77.6	34	9.3
P4	6 Jan 2025 – 13 Apr 2025	2,013	282.7 (223.5–379.5)	36.9	5.7	75.6	34	8.6
P5	14 Apr 2025 – 30 Jun 2025	1,917	228.9 (168.2–332.8)	58.1	5.6	78.7	34	7.1

Periods were identified by change-point analysis of the weekly median log-transformed concentration; the number of periods was chosen by the Bayesian information criterion. They are not recorded reagent lots or calibration events.

Table S3 (continued).

B. Median serum biotin by calendar month and year

Month	2024, n	2024, median (IQR), ng/L	2025, n	2025, median (IQR), ng/L
January	519	231.7 (166.2–344.3)	732	273.1 (219.0–372.9)
February	462	255.7 (194.8–389.3)	659	273.6 (214.4–373.9)
March	476	255.2 (199.0–372.8)	528	290.5 (217.3–378.8)
April	442	306.3 (237.4–446.4)	623	259.6 (200.2–356.0)
May	619	306.3 (240.8–451.0)	807	221.4 (163.6–336.6)
June	532	317.9 (235.6–440.8)	691	225.6 (163.2–325.0)
July	868	316.3 (239.0–437.2)	—	—
August	902	302.3 (237.7–415.5)	—	—
September	781	301.8 (239.7–409.6)	—	—
October	795	228.7 (163.4–330.0)	—	—
November	626	241.1 (179.1–345.8)	—	—
December	672	240.0 (168.5–343.1)	—	—

The study window ended in June 2025. In the months sampled in both years, the monthly pattern differed between years (month × year interaction in censored regression adjusted for age, sex, and nationality: $p < 0.001$).

C. Geometric mean serum biotin by season (ng/L, 95% CI) from censored regression

Specification	Winter	Spring	Summer	Autumn	p (season)
**Complete calendar year 2024 (n = 7,693)**					
Unadjusted	274.0 (266.5–281.7)	330.2 (320.2–340.2)	340.4 (331.9–349.3)	281.3 (274.3–288.3)	<0.001
Adjusted for age, sex, and nationality	275.3 (267.9–283.2)	328.8 (319.5–339.0)	341.6 (333.2–349.8)	279.7 (272.8–286.7)	<0.001
+ data-derived period	307.4 (296.8–318.2)	318.4 (308.8–328.4)	304.9 (295.8–314.3)	296.5 (288.1–305.0)	0.024
**Pooled 18 months (n = 11,733)**					
Unadjusted	285.9 (280.1–291.9)	300.0 (293.9–306.3)	318.7 (311.8–325.5)	281.3 (274.3–288.4)	<0.001
Adjusted for age, sex, and nationality	286.6 (280.8–292.5)	299.2 (293.5–304.9)	319.6 (312.9–326.6)	280.0 (272.9–287.2)	<0.001
+ calendar year	290.1 (284.2–296.1)	306.2 (300.3–312.5)	315.7 (309.0–322.6)	269.8 (262.7–277.0)	<0.001
+ data-derived period (instead of calendar year)	295.3 (288.0–303.4)	303.7 (297.0–310.4)	296.1 (288.2–303.9)	290.4 (282.0–299.4)	0.13

p (season): Wald test of the three seasonal terms. CI = confidence interval. Models exclude one participant without a recorded sex. Estimates are standardized to the covariate distribution of the participants in each analysis; confidence intervals were obtained by simulation from the estimated coefficient covariance matrix (4,000 draws). Kruskal–Wallis test across seasons in the pooled data: $H = 97.1$, $p < 0.001$. Unadjusted pairwise comparisons between seasons in the

pooled data (Mann–Whitney U test, Bonferroni-corrected): winter vs spring, $p = 0.001$; winter vs summer, $p < 0.001$; winter vs autumn, $p = 1.00$; spring vs summer, $p < 0.001$; spring vs autumn, $p = 0.004$; summer vs autumn, $p < 0.001$.

Table S3 (continued).

D. Median serum biotin by season in the complete calendar year 2024, overall and by subgroup (ng/L)

Group	n	Winter	Spring	Summer	Autumn	p*
All participants	7,694	243.2	290.7	308.5	262.9	<0.001
Females	6,146	241.7	284.1	302.3	256.1	<0.001
Males	1,547	256.4	322.9	343.1	288.5	<0.001
18–34 years	3,960	227.7	272.9	289.1	245.9	<0.001
35–49 years	2,684	258.7	293.8	312.7	264.6	<0.001
≥50 years	1,050	294.7	376.9	395.7	320.8	<0.001
Saudi	7,055	242.1	287.2	305.7	260.7	<0.001
Non-Saudi	639	258.0	352.9	360.4	291.0	<0.001

* Kruskal–Wallis test across the four seasons (all participants: H = 277.0). One participant without a recorded sex is not included in the rows by sex.

Table S4. Regression models, including sex-specific, nonlinearity, and sensitivity analyses.

A. Binary logistic regression: result at the upper limit of the measuring range (≥1,100 ng/L)

Model and predictor	Odds ratio (95% CI)	p
**All participants (n = 11,733; 772 events)**		
Sex (female vs. male)	1.93 (1.55–2.40)	<0.001
Age (per 10 years)	1.07 (1.00–1.13)	0.042
Nationality (non-Saudi vs. Saudi)	1.38 (1.09–1.75)	0.007
**Females (n = 9,269; 675 events)**		
Age (per 10 years)	1.06 (0.99–1.13)	0.099
Nationality (non-Saudi vs. Saudi)	1.25 (0.96–1.62)	0.096
**Males (n = 2,464; 97 events)**		
Age (per 10 years)	1.10 (0.94–1.28)	0.22
Nationality (non-Saudi vs. Saudi)	2.57 (1.45–4.57)	0.001
**All participants, additionally adjusted for data-derived period**		
Sex (female vs. male)	1.89 (1.52–2.35)	<0.001
Age (per 10 years)	1.07 (1.00–1.13)	0.039
Nationality (non-Saudi vs. Saudi)	1.39 (1.09–1.76)	0.007

Age × sex interaction: p = 0.59. Departure of age from linearity (restricted cubic spline, likelihood-ratio test, 2 degrees of freedom): p <0.001. Reference categories: male sex, Saudi nationality.

Table S4 (continued).

B. Censored (Tobit) regression of log-transformed serum biotin concentration

Model and predictor	Difference, % (95% CI)	p
**All participants (n = 11,733)**		
Age (per 10 years)	9.0 (8.0 to 10.0)	<0.001
Sex (female vs. male)	−1.3 (−3.9 to 1.3)	0.32
Nationality (non-Saudi vs. Saudi)	11.4 (7.0 to 15.9)	<0.001
**Females (n = 9,269)**		
Age (per 10 years)	8.9 (7.7 to 10.1)	<0.001
Nationality (non-Saudi vs. Saudi)	9.5 (4.7 to 14.5)	<0.001
**Males (n = 2,464)**		
Age (per 10 years)	9.1 (7.3 to 10.9)	<0.001
Nationality (non-Saudi vs. Saudi)	20.2 (10.6 to 30.5)	<0.001
**All participants, additionally adjusted for data-derived period**		
Age (per 10 years)	9.0 (8.0 to 10.0)	<0.001
Sex (female vs. male)	−1.8 (−4.3 to 0.8)	0.17
Nationality (non-Saudi vs. Saudi)	12.4 (8.1 to 16.8)	<0.001
**Participants aged 18–65 years (n = 11,443)**		
Age (per 10 years)	8.6 (7.5 to 9.7)	<0.001
Sex (female vs. male)	−1.1 (−3.7 to 1.7)	0.44
Nationality (non-Saudi vs. Saudi)	11.5 (7.1 to 16.0)	<0.001

Age × sex interaction: $p = 0.82$. Departure of age from linearity (restricted cubic spline, likelihood-ratio test, 2 degrees of freedom): $p = 0.86$. Results at or above 1,100 ng/L were treated as right-censored. Ordinary least-squares models are given in Table 4 of the main text.

C. Median regression of log-transformed serum biotin concentration (n = 11,733)

Predictor	Difference in median, % (95% CI)	p
Age (per 10 years)	9.6 (8.7 to 10.6)	<0.001
Sex (female vs. male)	−7.6 (−9.9 to −5.3)	<0.001
Nationality (non-Saudi vs. Saudi)	10.7 (6.7 to 14.9)	<0.001

Median regression is unaffected by right-censoring, because the conditional medians lie below the upper limit of the measuring range. The censored model in part B assumes a log-normal distribution; more results were recorded at the upper limit than this distribution predicts, so its estimate for sex, which depends on the upper tail, should be read together with part C and with the sensitivity analysis in Table 4. Variance inflation factors for the three predictors did not exceed 1.01.

Table S5. Model-based estimates of serum biotin concentration for the results recorded at the upper limit of the measuring range (n = 772).

Results above the measuring range were recorded at its upper limit (1,100 ng/L), so their concentrations are not known. To indicate their possible magnitude, concentrations were estimated under two models. The estimates are not measured values.

Model A (log-normal upper tail). The censored (Tobit) regression of log-transformed concentration on age, sex, and nationality (Table 4 of the main text) defines, for each participant, a log-normal distribution. The estimate for a right-censored result is the part of that distribution at or above 1,100 ng/L; its median and its 2.5th and 97.5th percentiles are reported.

Model B (continued density). Between 600 and 1,100 ng/L the observed density of results was nearly flat. This density was continued above the limit as an exponential tail, separately by sex, with the density at the limit equal to the observed density between 600 and 1,100 ng/L and the area under the tail equal to the number of right-censored results. The median and the 2.5th and 97.5th percentiles of the tail are reported.

Model	Median (IQR), ng/L	90th percentile	97.5th percentile	Median, females	Median, males	Cohort mean, ng/L
Model A: upper tail of the censored log-normal regression	1,286 (1,176–1,485)	1,770	2,254	1,285	1,290	370.8
Model B: continuation of the density observed at 600–1,100 ng/L	1,666 (1,331–2,259)	3,064	4,302	1,724	1,400	407.3

The cohort mean with the upper-limit results taken as recorded is 351.9 ng/L; the cohort median (270.9 ng/L) is not affected by either model. Model B parameters: females, 375 results between 600 and 1,100 ng/L and 675 at the upper limit (mean excess above the limit 900 ng/L); males, 112 and 97 (mean excess 433 ng/L). The fitted log-normal model places 1.6% of results at or above the upper limit (observed 6.6%) and 10.8% between 600 and 1,100 ng/L (observed 4.2%). Model A is the upper tail of a model that does not fit the distribution above 600 ng/L, and model B depends on the choice of the 600–1,100 ng/L window and on the assumed exponential shape. The two models show how strongly the estimate depends on the assumption; neither is confirmed by measurement, and the true values could be higher than both if the upper-limit results reflect supplement use. Estimates by sex, age group, and nationality for each of the 772 results are given in the accompanying Excel file (Table S5).
